# Extraction of Soluble Dietary Fiber from Sunflower Receptacles (*Helianthus annuus* L.) and Its Alleviating Effect on Constipation in Mice

**DOI:** 10.3390/nu16213650

**Published:** 2024-10-26

**Authors:** Shengying Zhu, Min Yan, Yanjing Feng, Jiayi Yin, Siyu Jiang, Yulong Guan, Bo Gao

**Affiliations:** 1 School of Life Sciences, Jilin University, Changchun 130012, China; zhusy22@mails.jlu.edu.cn (S.Z.); yanmin@genscigroup.com (M.Y.); fengyj23@mails.jlu.edu.cn (Y.F.); jiayiy24@mails.jlu.edu.cn (J.Y.); jsy24@mails.jlu.edu.cn (S.J.); guanyl23@mails.jlu.edu.cn (Y.G.); 2 Changchun GeneScience Pharmaceutical Co., Ltd., Changchun 130013, China; 3 Key Laboratory for Molecular Enzymology and Engineering, Jilin University, Ministry of Education, Changchun 130012, China

**Keywords:** sunflower receptacles, soluble dietary fiber, extraction, physicochemical properties, animal experiment, laxative

## Abstract

Background/Objectives: Sunflower receptacles are the main by-product of the processing of *Helianthus annuus* L. Methods: In this study, several extraction methods of soluble dietary fiber (SDF) from sunflower receptacles were evaluated, and then, the physicochemical structure and functional properties of these SDFs were examined. Finally, a mouse constipation model was established to explore its therapeutic potential for constipation. Results: The results showed that the SDF yield of citric acid extraction and enzyme extraction was better than that of hot-water extraction. Structural characterization showed that the three SDF functional groups were similar and amorphous, while the surface distribution of the SDF obtained by the citric acid extraction method (ASDF) had more fine pores. Physicochemical analysis showed that ASDF had the best water-holding capacity, oil-holding capacity, and expansion force. Animal experiments showed that the first black stool defecation time of the model group changed significantly (*p* < 0.001), indicating that the model was successful. Compared with the model group, the middle- and high-dose groups reduced the first black stool defecation time (*p* < 0.05 or *p* < 0.01) and increased the fecal water content (*p* < 0.05). The high-dose group significantly promoted the intestinal peristalsis of mice (*p* < 0.05). From hematoxylin–eosin (H&E) staining, it can be seen that the three dose groups of ASDF can improve the damage of mouse colon tissue induced by loperamide hydrochloride to a certain extent. Conclusions: Our results show that ASDF has good physical and chemical properties and laxative properties and has broad development space in the field of health food.

## 1. Introduction

The sunflower (*Helianthus annuus* L.) is a widely cultivated economic crop and oil crop in the world. It is an important source of high-quality oil, protein, and dietary fiber [1,2,3]. Sunflower receptacles (SRs) are one of the by-products of the sunflower. For a long time, SR has been directly discarded or burned in situ after sunflower seed removal, resulting in environmental pollution and a waste of resources [4]. In recent years, due to its rich dietary fiber (DF) content, SR has been used in animal feed research and achieved certain results, but it is not suitable for extensive use because of its low protein content [5]. Therefore, this study intends to further develop the function of SR and extract its soluble dietary fiber, which is applicable to new products.

Constipation is a common functional gastrointestinal disease. There are many complex causes of constipation. Insufficient exercise, heredity, diet, intestinal flora, age, and gender can cause constipation [6,7]. The most common symptoms of constipation include reduced and difficult defecation, long duration, abdominal distension or pain, and in severe cases, can even lead to life-threatening irritable bowel syndrome and colon cancer [8]. The treatment of constipation currently includes non-drug intervention and drug therapy. In non-drug intervention, it includes increasing dietary fiber and water intake and lifestyle adjustments [9]. When these methods are difficult to work, drug therapy is needed. Various osmotic laxatives are commonly used in the treatment of constipation but may be ineffective and may be accompanied by adverse reactions [10]. Stimulating laxatives are commonly used to treat patients with chronic constipation, but long-term use leads to drug dependence, gastrointestinal tissue damage, and high recurrence rates [11]. Drugs approved by the Food and Drug Administration (FDA) for the treatment of constipation, such as rubiprostone, linalotide, and plecanatide, also have a range of adverse reactions [12]. In view of the limitations of the current clinical medication, the endeavor is to develop anti-constipation products with less gastrointestinal irritation, fewer adverse reactions, and a pure nature.

Dietary fiber is recognized as a beneficial nutrient for human health. Clinical studies have shown that a higher dietary fiber intake can help reduce the risk of several chronic diseases, such as coronary heart disease, colorectal cancer, and breast cancer [13,14,15,16]. In addition, dietary fiber intake can increase fecal volume, reduce transit time, and regulate defecation, fermentation, and the effects on gut microbiota to promote digestive system health [17]. According to the solubility, soluble fiber (SDF) and insoluble fiber (IDF) can be divided [14]. SDF mainly includes pectin, oligosaccharides, glucan, and galactooligosaccharides, while IDF mainly includes cellulose, hemicellulose, and lignin [18,19]. Studies have shown that SDF and IDF have different physiological functions. IDF will not be fermented in the colon, but it increases the volume of feces and promotes gastrointestinal motility through its water-holding capacity [19]. Different from IDF, SDF is more easily utilized by intestinal flora and can affect the types and richness of intestinal microorganisms in the body. Intake of a large amount of SDF can reduce the risk of many chronic distortions [20].

According to the 2020–2025 Dietary Guidelines for American Residents (DGA), more than 90% of women and 97% of men do not meet the recommended intake of dietary fiber [21,22]. The American Nutrition and Dietetic Association calls for daily dietary fiber intake to reach 25 g for adult women and 38 g for adult men [23]. It is noteworthy that SDF has been used in the treatment of functional gastrointestinal disorders (FGIDs) in recent years. A clinical study has shown that guar gum can be used as a non-drug therapy for pediatric FGIDs and has achieved good results [24]. Therefore, the development of dietary fiber products has broad market prospects. However, studies have found that SDF content is relatively low in most dietary fiber-derived foods [25]. Therefore, it is necessary to increase the SDF content in dietary fiber foods to improve the functional and nutritional properties of modified products.

In view of the limitations of the current clinical treatment of constipation, based on the current situation of SR utilization, this paper uses the citric acid extraction method and the cellulase extraction method to modify the extraction of soluble dietary fiber from sunflower plate on the basis of the hot-water extraction method and uses the response surface method to optimize the extraction parameters and obtain the optimal extraction process. Then, the physicochemical properties and functional properties of the three SDFs were evaluated, and the properties of SDFs obtained by different extraction processes were evaluated. Finally, a mouse constipation model was established, which provided a more theoretical basis for the study of SR functional food in the treatment of constipation, and also provided a new way for the transformation of the agricultural and sideline products of SR into treasures having potential application.

## 2. Materials and Methods

### 2.1. Materials

*Helianthus annuus* L. was harvested from Baicheng City, Jilin Province (122°50′ E, 45° 37′ N). The plant specimens were identified by Professor Shuwen Guan from the College of Life Sciences at Jilin University.

Cellulase, α-amylase, and neutral protease were purchased from Beijing Solarbio Science & Technology Co., Ltd. (Beijing, China). Loperamide hydrochloride capsules were purchased from Xi ‘an Janssen Pharmaceutical Co., Ltd. (Xi’an, China); all reagents are of analytical grade.

### 2.2. Samples and Sample Preparation

Freshly picked sunflowers were deseeded, and the SRs were naturally air-dried to a constant weight. The SR powder was crushed (34,000 r/min, 3 min) with a high-speed grinder (800Y, Yongkang Platinum Europe Co., Ltd., Yongkang, China). Petroleum ether (1:4, *w*/*v*) was added to the SR powder for overnight soaking and degreasing, vacuum filtration, and drying in a blast oven (DHG-9070A, Shanghai Yiheng Scientific Instrument Factory, Shanghai, China) at 45 °C to constant weight.

### 2.3. SDF Extraction

#### 2.3.1. Citric Acid Extraction

In order to prepare SDF by citric acid extraction, the SR powder was mixed with deionized water in a certain proportion, and a certain mass fraction of citric acid was added. At a certain temperature, the water bath (HH-8, Guohua Electric Co., Ltd., Jintan, China) was used for a certain time to obtain the extract. After cooling, the mixture was centrifuged (1840× *g*, 10 min) by a centrifuge (PK-165, Hunan Pingke Scientific Instrument Co., Ltd., Changsha, China) to obtain the supernatant, then concentrated to a certain volume at 50 °C using a rotary evaporator (RF-52AA, Shanghai Yarong Biochemical Instrument Co., Ltd., Shanghai, China). The concentrated solution was mixed with 4 times the volume of ethanol (95%, *v*/*v*) and rested overnight. The precipitate was collected by vacuum filtration and washed with ethanol, and then, a vacuum-drying oven (DZF-6030B, Shanghai Yiheng Scientific Instrument Factory, Shanghai, China) was used to vacuum-dry to a constant weight at 50 °C. The product was designated as ASDF.

#### 2.3.2. Hot-Water Extraction

In order to prepare the SDF by hot-water extraction, the SR powder was mixed with distilled water in a certain proportion, and the extract was obtained by water bath for a certain time at a certain temperature. After cooling, the pH was adjusted to 6.5, and 1% (*w*/*w*) α-amylase and neutral protease were added for enzymatic hydrolysis at 50 °C for 60 min. Subsequently, the enzyme was inactivated by a boiling water bath for 5 min. Then the method of solid–liquid separation and ethanol precipitation was used, which was similar to the method described by the citric acid extraction method (Section 2.3.1), and the obtained dietary fiber was expressed as WSDF.

#### 2.3.3. Enzymatic Extraction

In order to prepare SDF by enzyme extraction, the SR powder was mixed with deionized water in a certain proportion, and a certain mass fraction of cellulase (Cat#C8271, 50,000 U/g, Beijing Solarbio Science & Technology Co., Ltd., Beijing, China) was added to adjust the pH to 6.5. The extract was obtained by water bath at a certain temperature for a certain time. Subsequently, the enzyme was killed by a boiling water bath for 5 min. Then, the method described in Section 2.3.2 was used for further enzymatic treatment, and finally, the dietary fiber representing the ESDF was obtained.

### 2.4. Optimization of SDF Extraction Process

#### 2.4.1. Mono-Factor Experiments

The effects of solid–liquid ratio, extraction time, temperature, citric acid addition, and cellulase addition on the yield of SDF from SR were tested by a single-factor test. For ASDF, the extraction conditions were temperature (50, 60, 70, 80, and 90 °C), extraction time (30, 60, 90, 120, and 150 min), liquid–solid ratio (15, 20, 25, 30, and 35 mL/g), and citric acid addition (0.5, 1.0, 1.5, 2.0, and 2.5%). For the WSDF, the extraction conditions were temperature (50, 60, 70, 80, and 90 °C), extraction time (30, 60, 90, 120, and 150 min), and liquid–solid ratio (20, 25, 30, 35, and 40 mL/g). For the ESDF, the extraction conditions were temperature (45, 50, 55, 60, and 65 °C), extraction time (30, 60, 90, 120, and 150 min), liquid–solid ratio (15, 20, 25, 30, and 35 mL/g), and cellulase addition (0.5, 1.0, 1.5, 2.0, and 2.5%).

#### 2.4.2. Response Surface Experiments

According to the results of the single-factor experiment, the Box–Behnken experiment (BBD) was designed by Design-Expert 8, and then, different experimental extraction conditions were optimized by the response surface method. For hot-water extraction, the independent variables were the liquid–solid ratio (A, 30, 35, and 40 mL/g), extraction time (B, 90, 120, and 150 min), and temperature (C, 70, 80, and 90 °C), and the response index was the WSDF yield. For the citric acid extraction method, the independent variables were the liquid–solid ratio (C, 15, 20, and 25 mL/g), extraction time (B, 60, 90, and 120 min), temperature (A, 60, 70, and 80 °C), and citric acid addition (D, 0.5, 1.0, and 1.5 %), and the response index was the ASDF yield. For the cellulase extraction method, the independent variables were the liquid–solid ratio (A, 20, 25, and 30 mL/g), time (B, 60, 90, and 120 min), temperature (C, 50, 55, and 60 °C), and cellulase addition (D, 1.0, 1.5, and 2.0 %), and the response index was the ESDF yield. The center value is encoded as 0, and the maximum and minimum values are encoded as 1 and −1, respectively. For the hot-water extraction method, a total of 17 groups were designed for the experiment, and 5 groups were repeated for the central point experiment. A total of 29 groups were designed for the citric acid extraction method and the cellulase method, and 5 groups were repeated for the central point experiment. The experimental factors and level design are shown in Tables S1–S3.

#### 2.4.3. Extraction Yield

The calculation formula for the SDF extraction rate of sunflower receptacles is as follows:

Extraction yield(%)=(M1/M0)×100%

where M1 is the dry weight (g) of the SDF powder extracted from sunflower receptacles, and M0 is the dry weight (g) of the sunflower receptacles powder used for extraction.

### 2.5. Analysis of Physicochemical Property

#### 2.5.1. Measurement of Water-Holding Capacity (WHC)

According to the method [26], slightly modified, 1.00 g of the SDF obtained by the different extraction methods was accurately weighed in a centrifuge tube, and 40 mL deionized water was added, shaken well, and balanced at 25 °C for 12 h. Then, it was centrifuged at 1840× *g* for 15 min, and the weight of the sample after discarding the supernatant was weighed. The water-holding capacity is calculated according to the following formula:

$$WHC\left(g/g\right)=(M2-M0)/M0\times 100\%$$

where M2 is the mass of the sample after water absorption (g); M0 represents the initial mass (g) of the sample.

#### 2.5.2. Measurement of Oil-Holding Capacity (OHC)

A slightly modified method [27] was used to determine the oil-holding capacity. Accurately weigh 1.00 g of the SDF obtained by different extraction methods in a centrifuge tube, add 12 mL peanut oil, and mix at 4 °C for 1 h. Then, it was centrifuged at 1840× *g* for 15 min, and the weight of the sample after discarding the supernatant was weighed. The oil-holding capacity calculation formula is as follows:

$$OHC\left(g/g\right)=(M3-M0)/M0\times 100\%$$

where M3 is the mass of the sample after oil absorption (g); M0 represents the initial mass (g) of the sample.

#### 2.5.3. Measurement of Swelling Capacity (SC)

A total of 0.25 g of the SDF samples obtained by different extraction methods were accurately weighed in an expansion tube. After recording the scale (V1), 10 mL of distilled water were added. The mixture was fully shaken and stood at room temperature for 12 h to record the corresponding scale (V2) of the SDF after water absorption:

SC(mL/g)=(V2−V1)/M0×100%

where V1 is the volume (mL) of SDF before water absorption and expansion; V2 is the volume (mL) of SDF after water absorption and expansion; and M0 is the mass (g) of SDF before swelling.

### 2.6. Structural Characterization of Sunflower Receptacles’ Soluble Dietary Fiber

#### 2.6.1. Scanning Electron Microscopy (SEM) Analysis

The microstructure of SDF obtained by different extraction methods was observed by scanning electron microscopy (JSM-6701F, JEOL, Tokyo, Japan). The samples were fixed and sprayed with gold, and the parameters were set to 5.0 kV acceleration voltage at 5000 times magnification.

#### 2.6.2. Fourier Transform Infrared (FT-IR) Spectroscopy Analysis

The SDFs were fully mixed with KBr (1:100, *w*/*w*), fully ground in a quartz mortar and pressed into a transparent sheet with an appropriate thickness. The FT-IR spectrometer (Nicolet 6700, Thermo Fisher Technologies, Waltham, MA, USA) was used to scan at 400~4000 cm^−1^ wavenumbers.

#### 2.6.3. X-Ray Diffraction (XRD) Analysis

The SDFs obtained by different extraction methods were compared and analyzed by an X-ray diffractometer (Kratos AXIS SUPRA, Bruker Technology Co., Ltd., Karlsruhe, Germany). The voltage was 40 kV. The current was 20 mA. The X-ray wavelength was λ = 0.156 nm, and the scanning range was 5°–70°.

### 2.7. Animal Experiment

#### 2.7.1. Animals and Their Experiments

All procedures involving animal experiments have been approved by the Animal Ethics Committee of Jilin University. The experimental animals were raised in the Animal Experimental Center of the College of Life Sciences, Jilin University (SPF grade).

SPF male BALB/c mice (18–22 g, 4–6 weeks) were purchased from Liaoning Changsheng Biotechnology Co., Ltd. The experimental animals were not genetically modified during the experiment. The mice were accommodated in the Jilin University Experimental Animal Platform and were housed in different units at a temperature of 23 ± 1 °C and a relative humidity of 40~60%. Good ventilation and light–dark cycle were ensured. Unrestricted food and water were provided during the experiment. During the experiment, the physiological status and body weight of each group of mice were monitored every day, and the daily food intake and water intake of each cage of mice were calculated.

After successful modeling, the control group and the model group were given normal saline 10 mL/kg, and the low-dose group, the middle-dose group, and the high-dose group were given ASDF 0.25 g/kg, 0.5 g/kg, and 1 g/kg, respectively. The dose was 0.1 mL/10 g, with continuous gavage for 8 days twice a day.

#### 2.7.2. Constipation Model Induced by Loperamide Hydrochloride

After one week of adaptive growth, 35 mice were randomly divided into 5 groups, namely the control group, model group, ASDF low-dose group, ASDF medium-dose group, and ASDF high-dose group (n = 7 per group). The blank group was given normal saline 10 mL/kg by gavage, and the other groups were given 10 mL/kg loperamide hydrochloride solution (loperamide hydrochloride dissolved in normal saline, 1 mg/mL) by gavage. The dosage was 0.1 mL/10 g once a day for 16 consecutive days, and modeling was performed [28].

#### 2.7.3. Evaluation of Defecation Function of Constipated Mice

After the animal experiment modeling and the end of treatment, the first black stool discharge time of the mice was measured. Before the experiment, each group of mice was fasted for 16 h, and the mice were free to drink water during the fasting period. The blank group did not undergo any treatment, and each mouse in the other groups was intragastrically administered with loperamide hydrochloride (10 mL/kg) at a dose of 0.1 mL/10 g. After 30 min, all mice were given 0.2 mL of ink by gavage, and the first black stool discharge time, the number of fresh feces discharged within 4 h, and the wet weight of each mouse were recorded. After that, the feces were placed in a blast-drying oven and dried at 60 °C to a constant weight. The moisture content of the feces was calculated according to the following formula:

Fecal moisture content(%)=(M5−M4)/M5×100%

where M4 is the dry weight of the feces (g); m5 is the wet weight of feces (g).

It is worth noting that 5 g Arabic gum powder and 5 g activated carbon powder were added to 100 mL distilled water to prepare ink, and the water bath was heated and boiled, repeated many times, until the Arabic gum was completely dissolved, the bottom of the bottle was not precipitated, stored at room temperature, and oscillated evenly before each use.

#### 2.7.4. Small Intestine Propulsion Test

After the end of drug treatment, the intestinal propulsion rate of mice was measured. Before the experiment, the mice in each group were fasted for 16 h, and the mice were free to drink water during the fasting period. The blank group was not treated, and each mouse in the other groups was given loperamide hydrochloride (10 mL/kg) by gavage at a dose of 0.1 mL/10 g. After 30 min, all mice were given 0.2 mL of ink by gavage. After 30 min, the mice in each group were sacrificed by dislocation, and the entire intestine from the pylorus to the end of the colon was immediately collected. The total length of the small intestine and the length of the ink propulsion were measured. The small intestine propulsion rate was calculated as follows:

Intestinal propulsive rate(%)=L2/L1×100%

where L1 is the total length of the small intestine (cm), and L2 is the ink propulsion distance (cm).

#### 2.7.5. Histopathology

Part of the colon tissue was fixed in 4% paraformaldehyde for 24 h. The fixed colon tissue was embedded in paraffin and cut into 5 μm thin sections. The colon tissue sections were first dewaxed in xylene, rehydrated in gradient ethanol, and then observed with hematoxylin–eosin (H&E) staining. Histological analysis was performed, and images of colon tissue were observed under an optical microscope (magnified ×400, Nikon, Minato, Japan).

### 2.8. Statistical Analysis

SPSS18.0 statistical software was used for statistics, and the data were represented by “X ± SD”. One-way variance (ANOVA) was used for statistical analysis, and Tukey’s comparison test was used to obtain a significant difference (*p* < 0.05 was considered statistically significant, and highly significant differences were indicated at *p* < 0.01). Origin 2019b software and Graphpad Prism 8.0 software were used to make and process the charts.

## 3. Results

### 3.1. Optimization of SDF Extraction Process from Sunflower Receptacles

The optimal extraction process of three SDFs was obtained by single-factor extraction and response surface optimization (Figures S1–S3). The optimum conditions for the extraction process of the ASDF were as follows. The ratio of material to liquid was 21 mL/g. The extraction time was 96 min. The extraction temperature was 68 °C, and the added amount of citric acid was 1.0%. For the WSDF, the optimum conditions were as follows: a solid–liquid ratio of 34 mL/g, an extraction time of 119 min, and an extraction temperature of 81 °C. For the ESDF, the optimum extraction process was as follows. The solid–liquid ratio was 27 mL/g. The extraction time was 84 min. The extraction temperature was 56 °C, and the cellulase addition was 1.7% (Figures S4–S6).

### 3.2. Physicochemical Properties of SDF from Sunflower Receptacles

**Figure 1:**
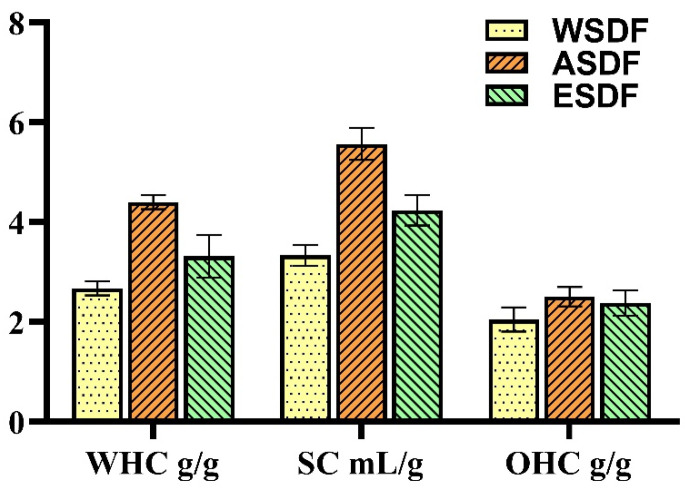
Physicochemical properties of soluble dietary fiber of sunflower receptacles (WSDF, ASDF, and ESDF). Results are expressed as mean ± SD (n = 3).

Water-holding capacity, swelling capacity, and oil-holding capacity are important physical and chemical properties that can reflect the quality of dietary fiber [29]. The water-holding capacity, swelling capacity, and oil-holding capacity of WSDF, ASDF, and ESDF are shown in Figure 1. The water-holding capacities of the three SDFs were 2.67 ± 0.11 g/g, 4.40 ± 0.11 g/g, and 3.31 ± 0.35 g/g. The expansion forces were 3.33 ± 0.17 mL/g, 5.57 ± 0.26 mL/g, and 4.23 ± 0.25 mL/g. The oil-holding capacities were 2.05 ± 0.20 g/g, 2.50 ± 0.16 g/g, and 2.38 ± 0.21 g/g, respectively. The above results show that ASDF has better water-holding capacity, swelling capacity, and oil-holding capacity than WSDF and ESDF.

### 3.3. Structural Analysis

**Figure 2:**
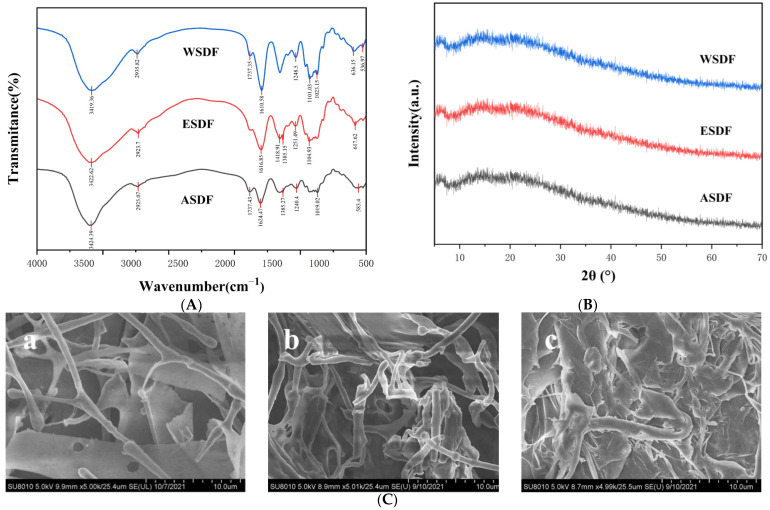
Structural characterization of SDF extracted by different methods. (**A**) FT-IR spectrum of soluble dietary fiber of sunflower receptacles (ASDF, ESDF, and WSDF). (**B**) X-ray diffraction of soluble dietary fiber of sunflower receptacles (ASDF, ESDF, and WSDF). (**C**) SEM images of soluble dietary fiber of sunflower receptacles, (**a**) WSDF, (**b**) ESDF, and (**c**) ASDF.

Infrared spectroscopy can effectively identify various characteristic functional groups in substances [30]. Figure 2A shows the Fourier transform infrared spectra of the SDF obtained by different extraction methods. According to the spectral analysis, their infrared absorption spectra are similar, indicating that the three SDFs have similar structural characteristics. There is a strong broad peak at about 3400 cm^−1^, which is attributed to the O-H stretching vibration of pectin and hemicellulose [31]. The characteristic absorption peak near 2930 cm^−1^ is caused by the C-H stretching vibration of the methyl and methylene groups in the polysaccharide [32]. The absorption peak between 1735 cm^−1^ and 1600~1650 cm^−1^ is the stretching vibration of C=O, which is for the C=O in the semi-cellulose ester group and the ester bond of the COOH group in the pectin, respectively [33,34,35]. The absorption peak at about 1420 cm^−1^ is C-H stretching vibration, and the absorption peak at 1320~1210 cm^−1^ is O-H variable-angle vibration. The absorption peak between 1000 and1200 cm^−1^ is the vibration of the glycosidic bond, indicating that there may be a pyranose ring [36]. The SDFs from three sunflower receptacles have similar functional groups, but the peak intensity is slightly different. The intensity difference at 1600~1650 cm^−1^ is larger, which may be due to the different uronic acid content of SDF.

XRD can analyze the crystal type and structural characteristics of SDF samples obtained by different extraction methods [37]. It can be observed from Figure 2B that there was almost no absorption peak in the three SDFs, and there was no significant difference in the crystal structure of the SDF extracted by these three methods. The results showed that the main structure of several SDF samples was sn amorphous structure, which was consistent with the typical characteristics of SDF [31].

Scanning electron microscopy was used to observe the surface morphology of the SDF obtained by different extraction methods (Figure 2C). From Figure 2C-a, we found that the WSDF extracted by hot-water extraction showed a coexistence of irregular filaments and flakes. From Figure 2C-b, it can be seen that the ESDF obtained by cellulase extraction presents an irregular fiber column, which may be due to the aggregation of many molecules or molecular groups into different patterns of bundles. Figure 2C-c shows that the ASDF obtained by citric acid extraction is a serrated sheet shape with many small pores on the surface. The results showed that different extraction methods had a great influence on the apparent morphology of SDF, and the surface of ASDF has more small pores than that of ESDF and WSDF, which may be one of the reasons why ASDF has a stronger water-holding capacity and expansion force.

### 3.4. Growth of Mice

In the whole experiment, all mice were in good condition, and no hair removal, fights, or death occurred. During the experiment, the body weight of the mice in each group gradually increased, but there was no significant difference between the groups. The average water intake and food intake of each group of mice were similar to those before modeling, which indicated that the dose and time of loperamide hydrochloride modeling would not cause serious damage to the health of the mice.

### 3.5. The Effect of Sunflower Receptacles ASDF on Mice Defecation

**Figure 3:**
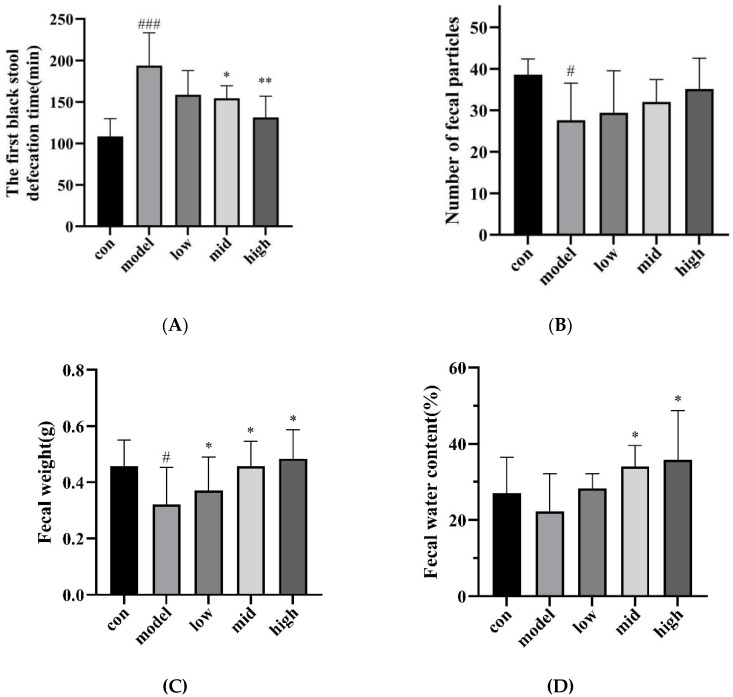
Mice defecation experiment results. (**A**) The time of the first black-stool defecation in mice after administration. (**B**) The number of fecal particles at 4 h after administration. (**C**) Fecal weight at 4 h after administration. (**D**) Fecal water content at 4 h after administration. Results are expressed as mean ± SD (n = 7). ^#^ is significantly different from the control group (*p* < 0.05), ^###^ was significantly different from the control group (*p* < 0.001); * Compared with the model group, the difference was significant (*p* < 0.05), ** Compared with the model group, the difference was significant (*p* < 0.01).

After 16 days of intragastric administration of loperamide hydrochloride, the first black-stool defecation time of the model group was significantly higher than that of the blank group (*p* < 0.001), which verified the effectiveness of loperamide hydrochloride modeling. After a period of treatment with ASDF, compared with the model group, the middle-dose group of ASDF significantly reduced the first black-stool defecation time (*p* < 0.05), while the high-dose group significantly reduced the first black-stool defecation time (*p* < 0.01). Although there was no significant difference in the low-dose group (*p* > 0.05), it still reduced part of the first black-stool discharge time (Figure 3A). The above results show that different doses of ASDF can alleviate constipation in mice to varying degrees, and the high-dose group has the best effect.

From Figure 3B, it can be seen that there was no significant difference in the number of defecation particles in mice at 4 h after administration compared with the model group (*p* > 0.05), but with the continuous increase of gavage dose, the number of defecation particles in mice increased. However, Figure 3C shows that, compared with the model group, the fecal weight of mice at 4 h after administration was significantly increased in the low, medium, and high ASDF dose groups (*p* < 0.05). In addition, in terms of fecal moisture content, when compared with the model group, the differences between the medium and high ASDF dose groups were significant (*p* < 0.05). Although there was no significant difference in the low ASDF dose group (*p* > 0.05), the moisture content was still higher than in the model group, which was equivalent to the moisture content of the control group (Figure 3D). The above results showed that the frequency of defecation, the weight of defecation, and the moisture content of the feces increased after the constipation mouse model was given different doses of ASDF. The weight of feces in the medium-dose ASDF group was comparable to that in the healthy group, while the weight of feces in the high-dose ASDF group was higher than that in the healthy group. In terms of fecal moisture content, the moisture content of the low, medium, and high ASDF dose groups exceeded that of the healthy group. Compared with the mice in the model group, the constipation symptoms of the treatment group have been alleviated to some extent. The effect on the high-dose group was better.

### 3.6. The Effect of Sunflower Receptacles ASDF on Intestinal Propulsion of Mice

**Figure 4:**
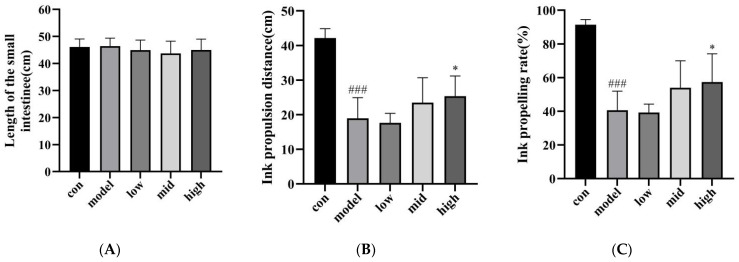
Experimental results of small intestine propulsion in mice. (**A**) Total length of small intestine in mice. (**B**) Ink propulsion distance. (**C**) Ink propelling rate. Results are expressed as mean ± SD (n = 7). ^###^ is significantly different from the control group (*p* < 0.001); * Compared with the model group, the difference was significant (*p* < 0.05).

It can be seen from Figure 4A that, compared with the blank group, there was no significant difference in the total length of the small intestine between the model group and the low, medium, and high ASDF dose groups (*p* > 0.05), indicating that the constipation model induced by loperamide hydrochloride did not affect the intestinal length of the mice. In terms of the ink propulsion distance and the intestinal propulsion rate in mice, compared with the model group, the ink propulsion distance and propulsion rate in the high-dose ASDF group were significantly increased (*p* > 0.05), and there was no significant difference in the medium-dose ASDF group (*p* > 0.05). However, compared with the model group, the ink propulsion distance and propulsion rate increased slightly (Figure 4B,C). After the successful establishment of the constipation model, the mice were intragastrically administered with different doses of sunflower disc ASDF. Compared with the model group, the ink propulsion distance and ink propulsion rate of the two groups of mice in the medium-dose and high-dose ASDF groups increased, and the two indicators in the high-dose ASDF group were significantly higher than those in the model control group (*p* < 0.05). It shows that the high-dose ASDF group has a better recovery effect on the intestinal propulsion ability of constipation mice.

### 3.7. Analysis of H&E Staining Results of Colon

**Figure 5:**
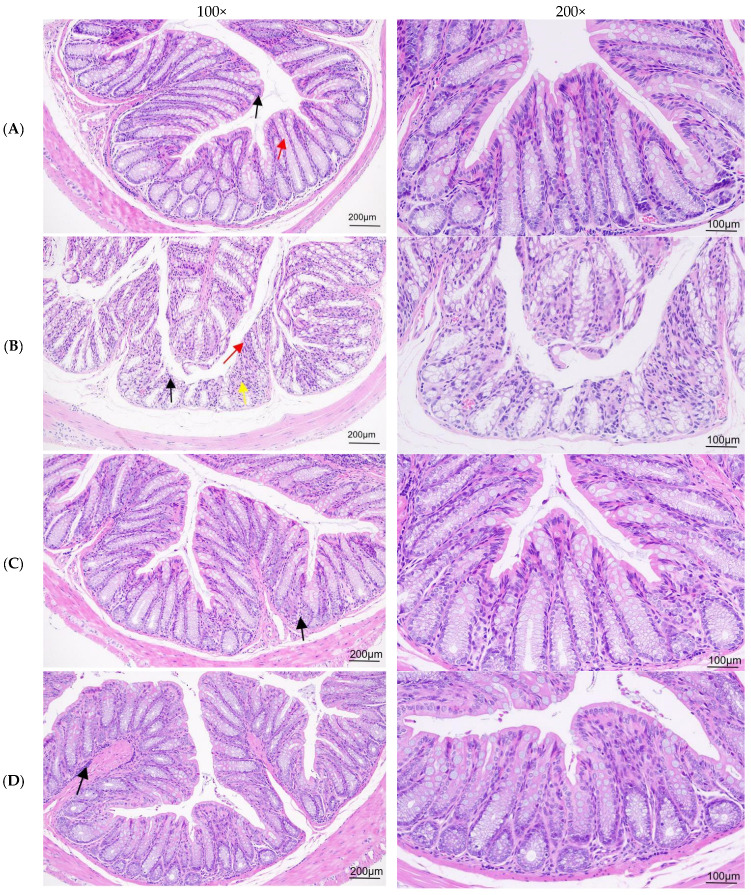

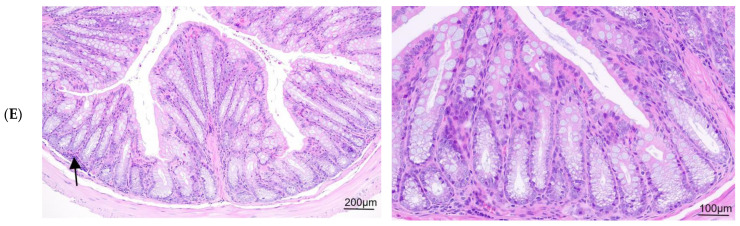
H&E staining results of mice colon. (**A**) Control group. (**B**) Model group. (**C**) ASDF low-dose group. (**D**) ASDF medium-dose group. (**E**) ASDF high-dose group. The black arrows indicate epithelial cells, the red arrows signify goblet cells, and the yellow arrows denote fibrous tissue hyperplasia.

The results of H&E staining of the colon are shown in Figure 5. Among them, it can be seen from Figure 5A of the blank group that the colon tissue structure is normal, the mucosal crypts are closely arranged, the goblet cells are abundant, the epithelial cells are not denatured and exfoliated, and there is no inflammatory cell infiltration in the tissue. The epithelial cells are shown as black arrows. Red arrows represent goblet cells. Figure 5B shows that, compared with the blank healthy group, the colon tissue structure of the model group changed abnormally, the number of mucosal crypts in some areas decreased significantly, the mucosal epithelial cells fell off, and the lamina propria were exposed, as shown by the red arrow. The number of crypts and goblet cells decreased, and fibrous tissue hyperplasia was seen, as shown by the yellow arrow. Inflammatory cell infiltration can be seen in the mucosal layer, as shown by black arrows. Compared with the model group, the tissue structure of the colon was basically normal in the low-dose group. The mucosal crypts were closely arranged, and the number was not reduced. There was no obvious degeneration and shedding of epithelial cells, and a small amount of inflammatory cell infiltration was observed, as shown by black arrows (Figure 5C). From Figure 5D, the staining of the middle-dose group was compared with that of the model group. The structure of the colon tissue recovered well. The crypts of the mucosa were arranged neatly, and the number was not reduced. No degeneration and shedding of epithelial cells were observed. A small amount of inflammatory cell infiltration was observed in the submucosa, as shown by black arrows. In Figure 5E, compared with the model group, the colonic tissue structure of the high-dose group basically returned to normal, the mucosal crypts were arranged neatly, the number was not reduced, the epithelial cells were not denatured and exfoliated, and a small amount of inflammatory cell infiltration was observed in the submucosa, as shown by black arrows. Through the HE staining results of the mouse colon, it can be seen that the three doses of ASDF have a certain repair function on the destruction of colon tissue caused by constipation and can play a role in protecting the colon.

## 4. Discussion

Soluble dietary fiber has been proven to have a series of functions, such as strong oil holding, water holding, expansion, induction of intestinal microorganisms, detoxification, and increase of intestinal peristalsis [38]. It can be used to prevent and treat constipation [39], reduce blood lipid [40], and prevent cancer [41]. Therefore, more and more scholars at home and abroad are studying it. At present, the raw materials for the preparation of SDF are mainly concentrated in wheat bran [42], rice bran [43], bean dregs [44], apple pomace [45], sugar beet pulp [35,46], etc., and the resources of SDF need to be further developed. SR is a by-product of sunflower production and processing. It is often abandoned in the field in sunflower-producing areas, causing serious pollution to the environment [4]. Moreover, the content of SDF in sunflower receptacles is low, which limits its application in improving intestinal function.

At present, SDF extraction methods mainly include chemical, physical, and biological technologies. Among them, chemical and enzymatic methods are more commonly used because of their simple operation, low energy consumption, and easy availability of materials [32]. The chemical method is considered to be an important method for improving the solubility of dietary fiber, and it is easy to increase the SDF yield [47]. Among them, acid extraction can effectively hydrolyze hemicellulose, thereby changing the composition of SDF and IDF [48]. In contrast, enzymatic extraction can destroy cellulose, hemicellulose, and lignin in the cell wall and promote the conversion of IDF into SDF [49]. The results of response surface optimization showed that the yield of WSDF in hot-water extraction was 13.17 ± 0.21 %, while the yield of ASDF obtained by citric acid extraction was 13.64 ± 0.27%, and the highest yield obtained by cellulase extraction was 15.29 ± 0.22%, which was higher than the WSDF. Therefore, compared with the water extraction method, both the citric acid extraction method and the cellulase extraction method could improve the extraction rate of SDF from sunflower receptacles, which was similar to other studies [32,50].

Different extraction methods can not only change the yield of SDF but also change the composition and structural properties of SDF and, then, change its physicochemical properties and functional properties [51]. Although SDF can also be obtained by hot-water extraction and cellulase extraction, our study found that the ASDF obtained by citric acid extraction was significantly superior to that from other extraction methods in terms of water-holding capacity, oil-holding capacity, and swelling capacity. Structural analysis shows that the excellent physical and chemical properties of ASDF can be attributed to its improved microstructure, which enables it to capture more water and oil molecules [52]. In addition, Wang et al. also pointed out that the SDF extracted by citric acid has a high water-holding capacity and glucose-adsorption capacity [34]. Due to the high water-holding capacity and swelling force of SDF, it usually can play a good laxative effect [53]. Given that our previous studies have shown that ASDF has good water-holding capacity and swelling power, it can be used as a material for subsequent research on laxative function.

Clinical studies have found that an important cause of constipation is abnormal digestive system function, increased stool transport time, and excessive intestinal absorption of food moisture, which makes the stool hard and difficult to excrete [54]. Dietary fiber is often used as a fecal water carrier to increase the volume of feces, which can effectively alleviate constipation by increasing the volume of feces [55]. With in vivo experiments, we established a mouse constipation model and treated it with citric acid-extracted ASDF. The results showed that the high-dose SDF group was significantly improved in various constipation indicators compared with the model group. Specifically, the first black-stool discharge time was shortened, the fecal water content was increased, and the small intestine peristalsis was enhanced, indicating that SDF can improve constipation by increasing the water content in the colon and stimulating peristalsis. In addition, compared with other dietary fibers, such as okara fiber, sunflower receptacle ASDF also played a good role in the first black-stool discharge time and fecal moisture content [56]. The results of H&E staining showed that the three dose groups of ASDF could improve the damage of colon tissue in constipation mice to a certain extent. The results indicate that ASDF not only contributed to the mechanical passage of feces but also had a protective effect on the inner wall of the gastrointestinal tract. The above results showed that ASDF could relieve constipation in mice, and the therapeutic effect showed a significant dose–effect relationship. These findings are particularly important given that modern consumers are increasingly pursuing drugs with natural and healthy ingredients [57]. Compared with the limitations of current clinical medication for constipation, the results of this study showed the safety and efficacy of sunflower receptacle soluble dietary fiber in the treatment of constipation. It reveals the great potential of the ASDF extracted from agricultural by-product sunflower receptacles in the treatment of constipation and provides a natural alternative therapy for the treatment of clinical constipation.

## 5. Conclusions

In summary, this study first used three extraction methods to extract and modify the SDF of sunflower receptacles, obtained the optimal modified fiber extraction process through characterization and physical and chemical analysis, and then established a mouse model to explore its mechanism of moistening intestines and laxatives. Our results showed that ASDF showed the best water-holding capacity and swelling capacity in different extraction processes, which was related to its microstructure. This finding provides evidence for the correlation between the physicochemical properties and functional benefits of dietary fiber. In addition, in vivo experimental results showed that ASDF could exert good laxative properties and improve the damage of colon tissue in mice with constipation to a certain extent. Based on the above research, we found that ASDF can be safely applied for the treatment of constipation, and the research results can provide a theoretical reference for the high-value utilization and development of sunflower receptacles as a functional food.

## Data Availability

Data are contained within the article and Supplementary Materials.

## Supplementary Materials

The following supporting information can be downloaded at https://www.mdpi.com/article/10.3390/nu16213650/s1: Figures S1–S3. Single-factor experimental results of WSDF, ASDF, ESDF; Figures S4–S6. Response surface optimization process results of WSDF; Tables S1–S3. Response surface factor level design of WSDF, ASDF, ESDF.
