# Supplementary material for: Extraction of Soluble Dietary Fiber from Sunflower Receptacles (Helianthus annuus L.) and Its Alleviating Effect on Constipation in Mice
Source: Nutrients. 2024 Oct 26;16(21):3650. doi: 10.3390/nu16213650 (PMC11547490; doi:10.3390/nu16213650)
Supplement: Supplementary file 1 [file nutrients-16-03650-s001.zip › Figures S1-S3.pdf]

Supplementary Materials S1

Figure S1. Effect of citric acid extraction on the yield of ASDF. (A) The effect of extraction temperature on the yield of ASDF. (B) The effect of extraction time on the yield of ASDF. (C) The effect of liquid-solid ratio on the yield of ASDF. (D) The effect of citric acid addition on the yield of ASDF.

Figure S2. Effect of hot water extraction on the yield of WSDF. (A) The effect of extraction temperature on the yield of WSDF. (B) The effect of extraction time on the yield of WSDF. (C) The effect of liquid-solid ratio on the yield of WSDF.

Figure S3. Effect of cellulase extraction on ESDF yield. (A) The effect of extraction temperature on the yield of ESDF. (B) The effect of extraction time on the yield of ESDF. (C) The effect of liquid-solid ratio on the yield of ESDF. (D) The effect of cellulase addition on the yield of ESDF.

Figure S1

(A)

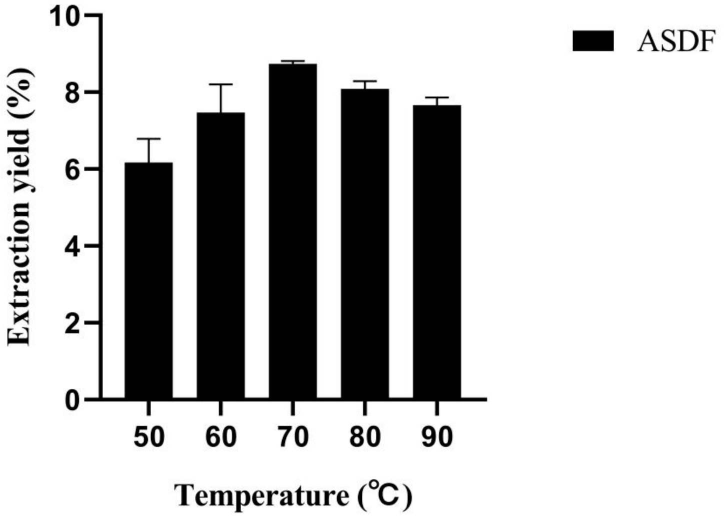

(B)

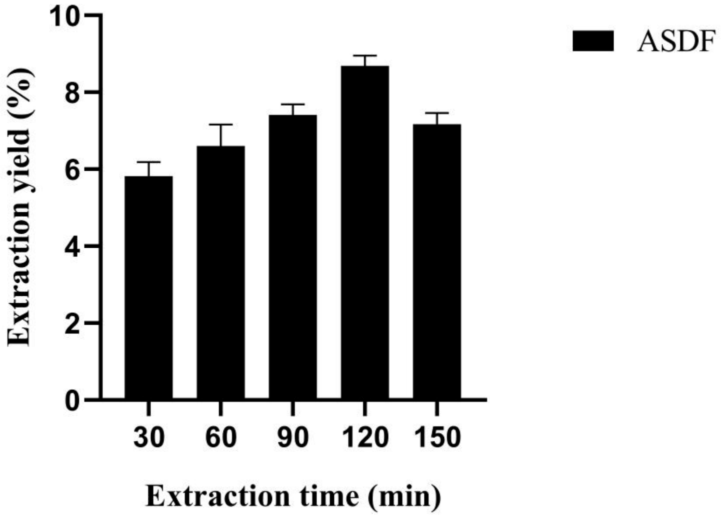

(C)

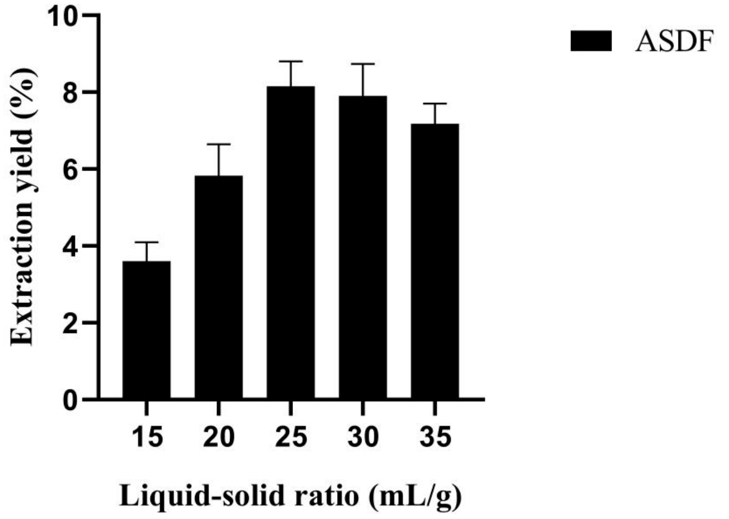
ASDF
Extraction yield (%)
10
8
6
4
2
0
15
20
25
30
35
Liquid-solid ratio (mL/g)

(D)

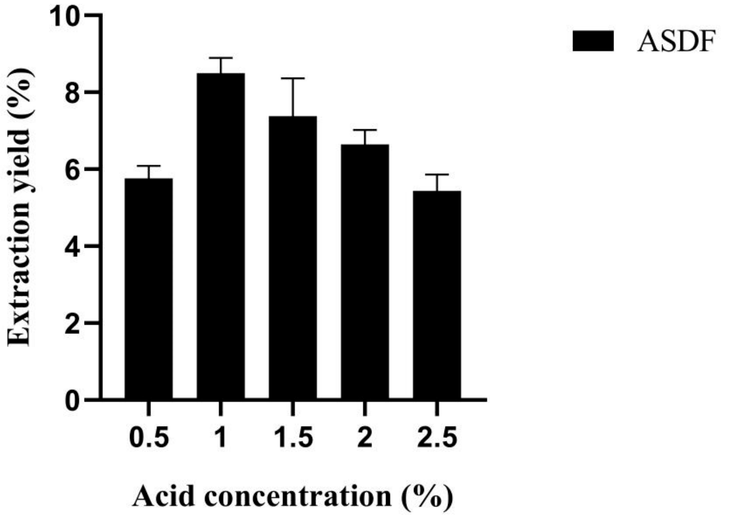
ASDF
Extraction yield (%)
10
8
6
4
2
0
0.5
1
1.5
2
2.5
Acid concentration (%)

Figure S2

(A)

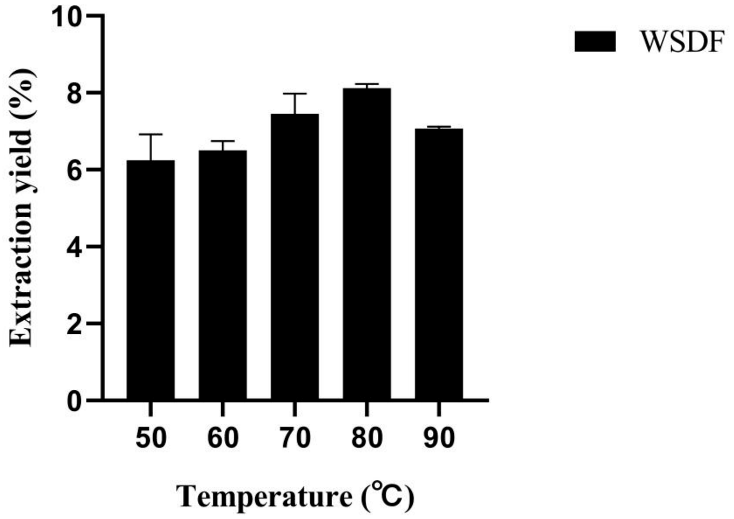

(B)

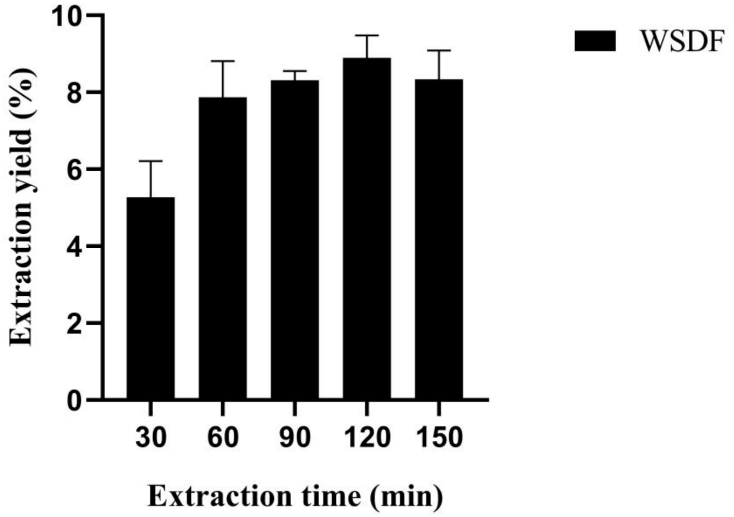

(C)

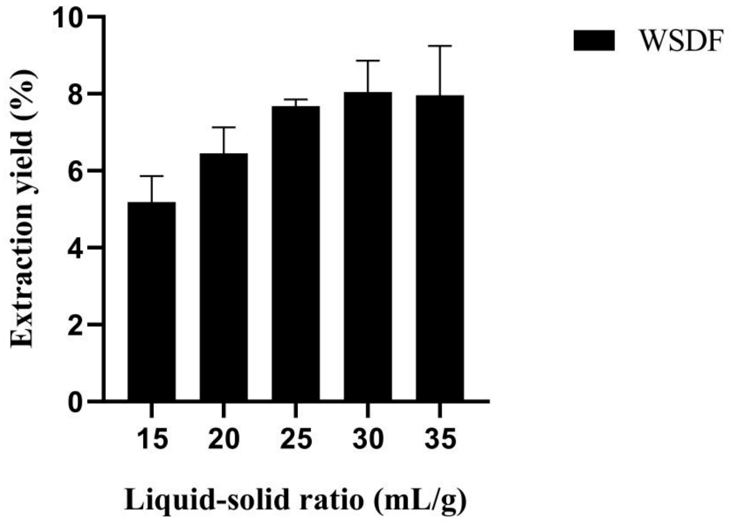
WSDF
Extraction yield (%)
10
8
6
4
2
0
15
20
25
30
35
Liquid-solid ratio (mL/g)

Figure S3

(A)

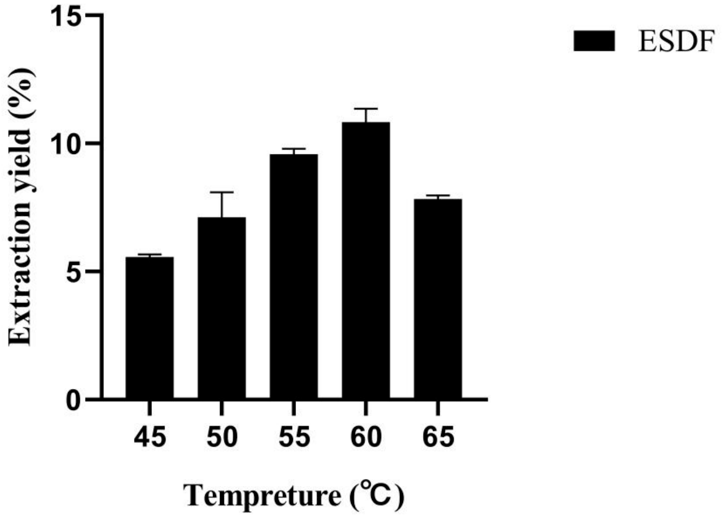
ESDF
Extraction yield (%)
15
10
5
0
45
50
55
60
65
Tempreture (℃)

(B)

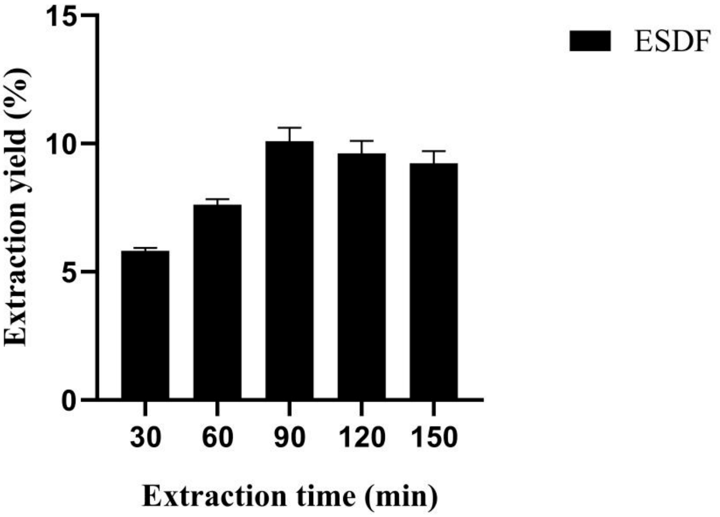
ESDF
Extraction yield (%)
0
5
10
15
30
60
90
120
150
Extraction time (min)

(C)

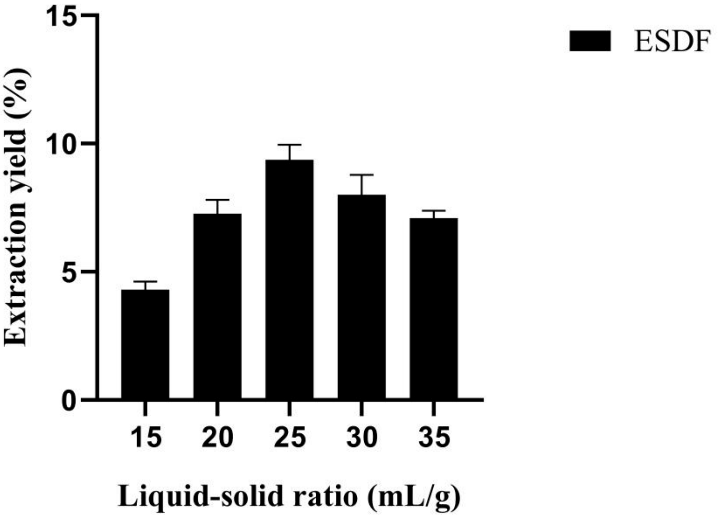
ESDF
Extraction yield (%)
0
5
10
15
15
20
25
30
35
Liquid-solid ratio (mL/g)

(D)

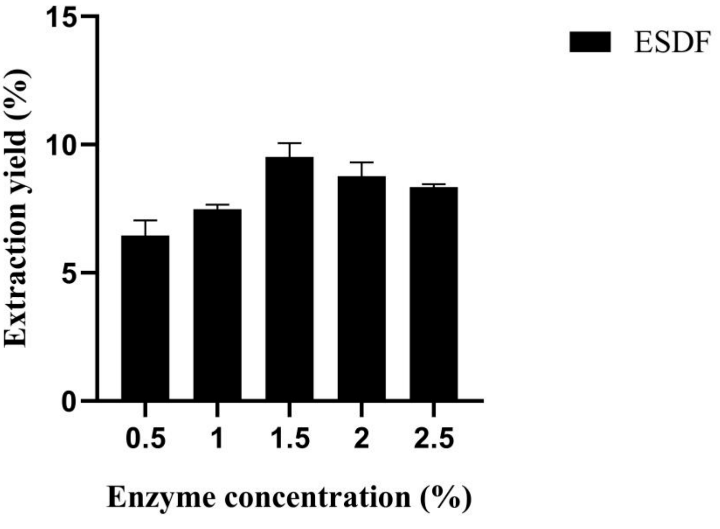
ESDF
Extraction yield (%)
0
5
10
15
0.5
1
1.5
2
2.5
Enzyme concentration (%)
