# Supplementary material for: Extraction of Soluble Dietary Fiber from Sunflower Receptacles (Helianthus annuus L.) and Its Alleviating Effect on Constipation in Mice
Source: Nutrients. 2024 Oct 26;16(21):3650. doi: 10.3390/nu16213650 (PMC11547490; doi:10.3390/nu16213650)
Supplement: Supplementary file 1 [file nutrients-16-03650-s001.zip › Figures S4-S6.pdf]

Supplementary Materials S3

Figure S4. The response surface plots of liquid-to-material ratio, extraction time, temperature and citric acid addition amount on the extraction of sunflower receptacles ASDF content optimization.

Figure S5. The response surface plots of liquid-to-material ratio, extraction time and temperature on the extraction of sunflower receptacles WSDF content optimization.

Figure S6. The response surface plots of liquid-to-material ratio, extraction time, temperature and cellulase addition amount on the extraction of sunflower receptacles ESDF content optimization.

Figure S4

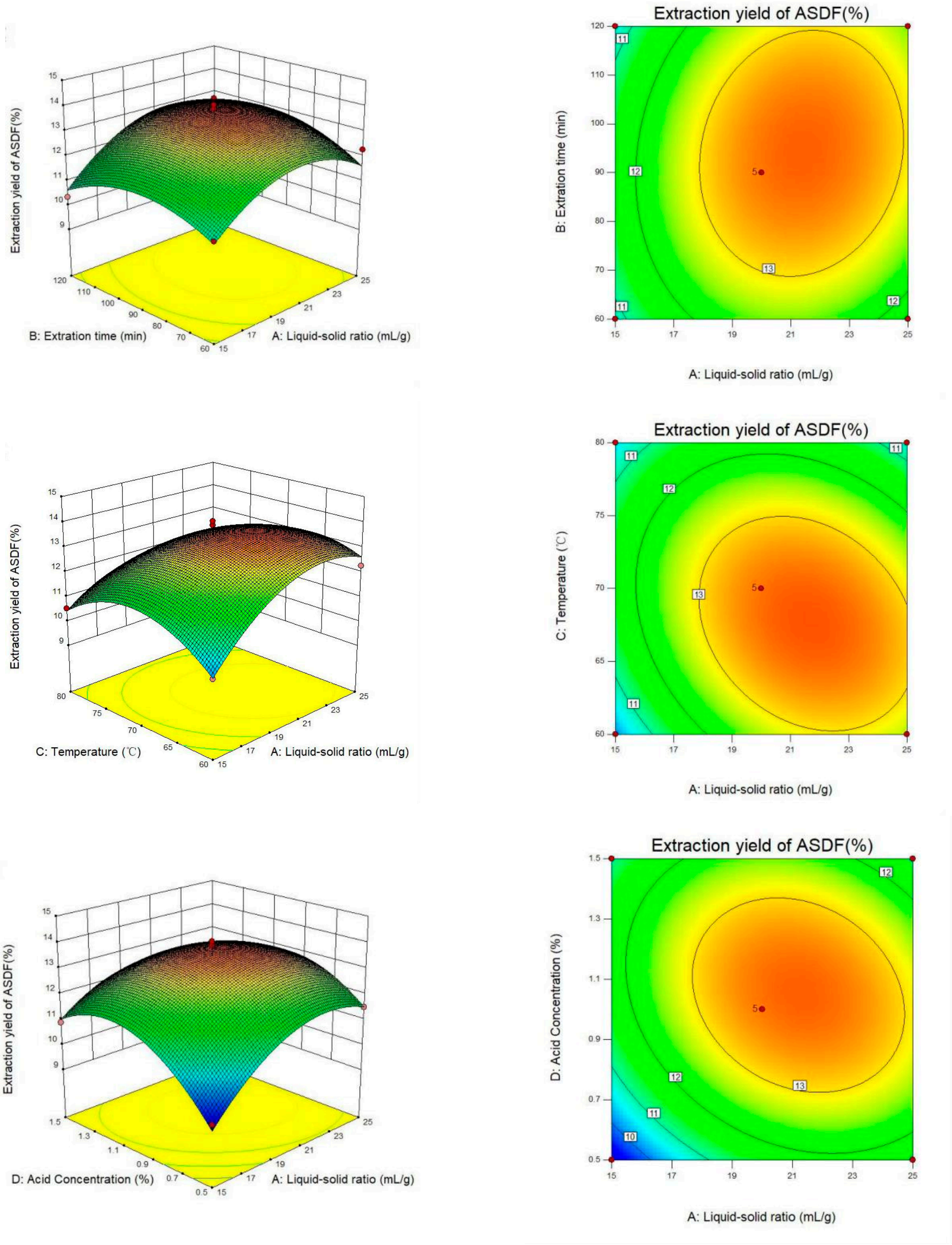

Extraction yield of ASDF(%)
B: Extration time (min)
A: Liquid-solid ratio (mL/g)
Extraction yield of ASDF(%)
B: Extration time (min)
A: Liquid-solid ratio (mL/g)
Extraction yield of ASDF(%)
C: Temperature (℃)
A: Liquid-solid ratio (mL/g)
Extraction yield of ASDF(%)
C: Temperature (℃)
A: Liquid-solid ratio (mL/g)
Extraction yield of ASDF(%)
D: Acid Concentration (%)
A: Liquid-solid ratio (mL/g)
Extraction yield of ASDF(%)
D: Acid Concentration (%)
A: Liquid-solid ratio (mL/g)

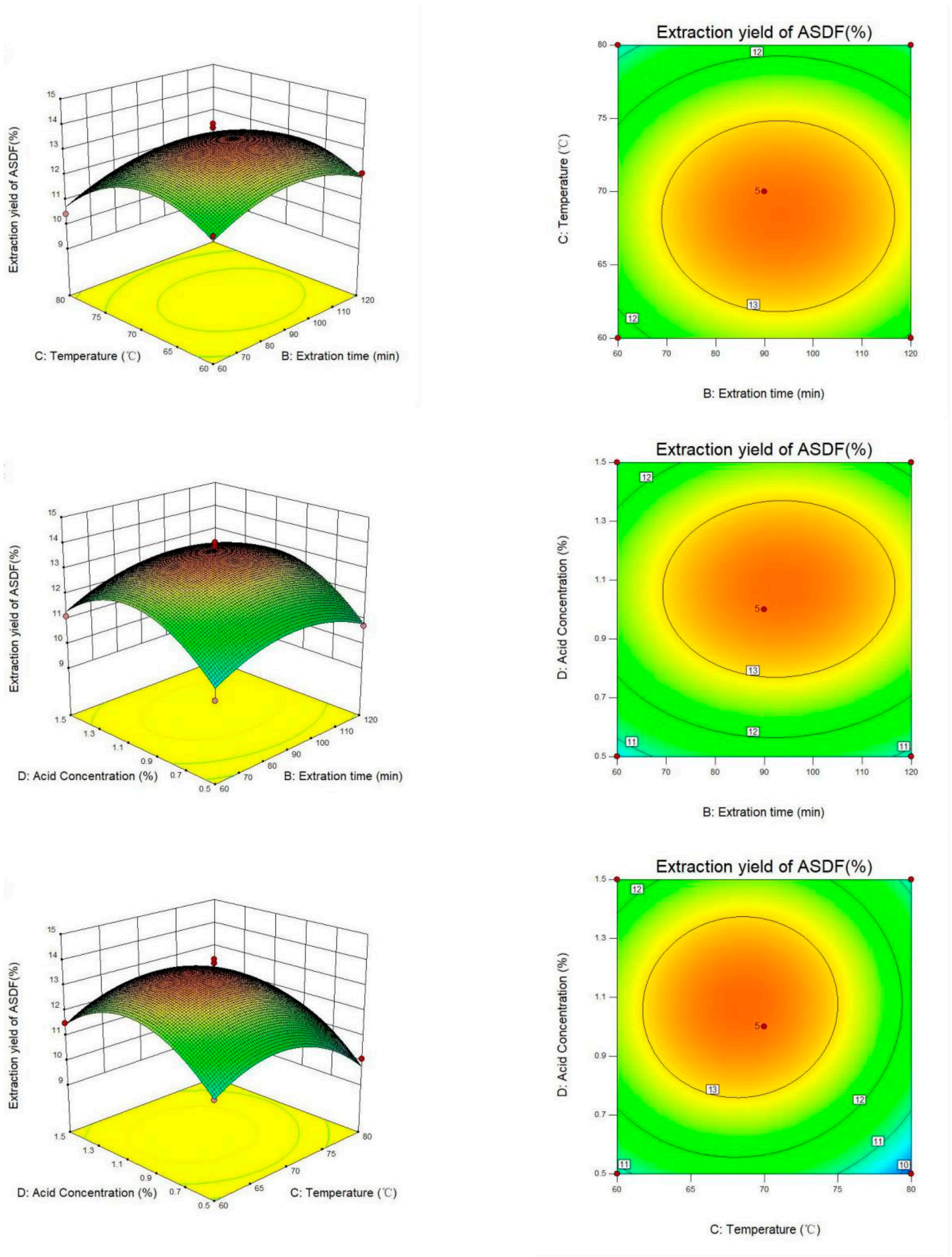
Extraction yield of ASDF(%)
C: Temperature (℃)
B: Extration time (min)
Extraction yield of ASDF(%)
D: Acid Concentration (%)
B: Extration time (min)
Extraction yield of ASDF(%)
D: Acid Concentration (%)
C: Temperature (℃)

Figure S5

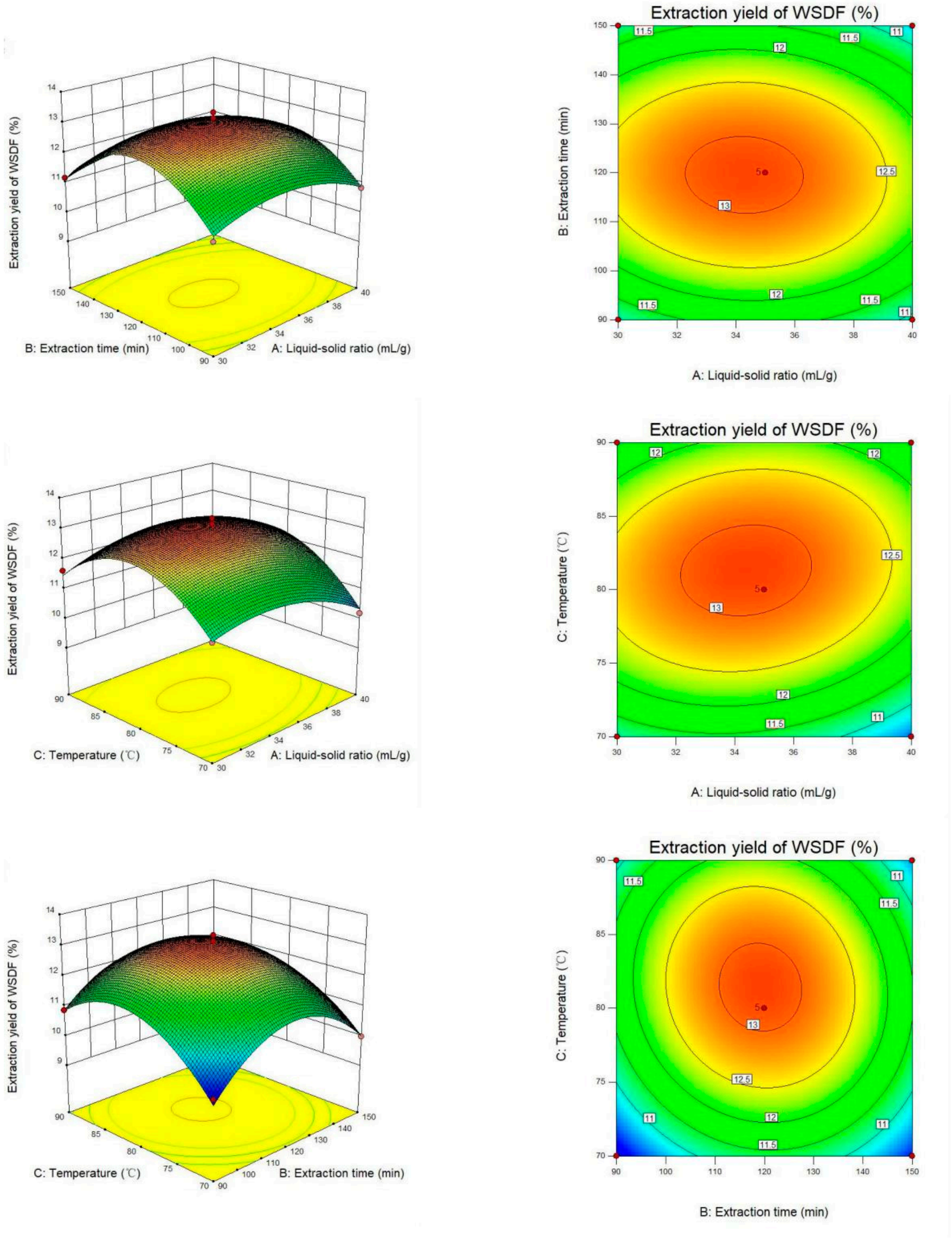
Extraction yield of WSDF (%)
B: Extraction time (min)
A: Liquid-solid ratio (mL/g)
Extraction yield of WSDF (%)
C: Temperature (℃)
A: Liquid-solid ratio (mL/g)
Extraction yield of WSDF (%)
C: Temperature (℃)
B: Extraction time (min)

Figure S6

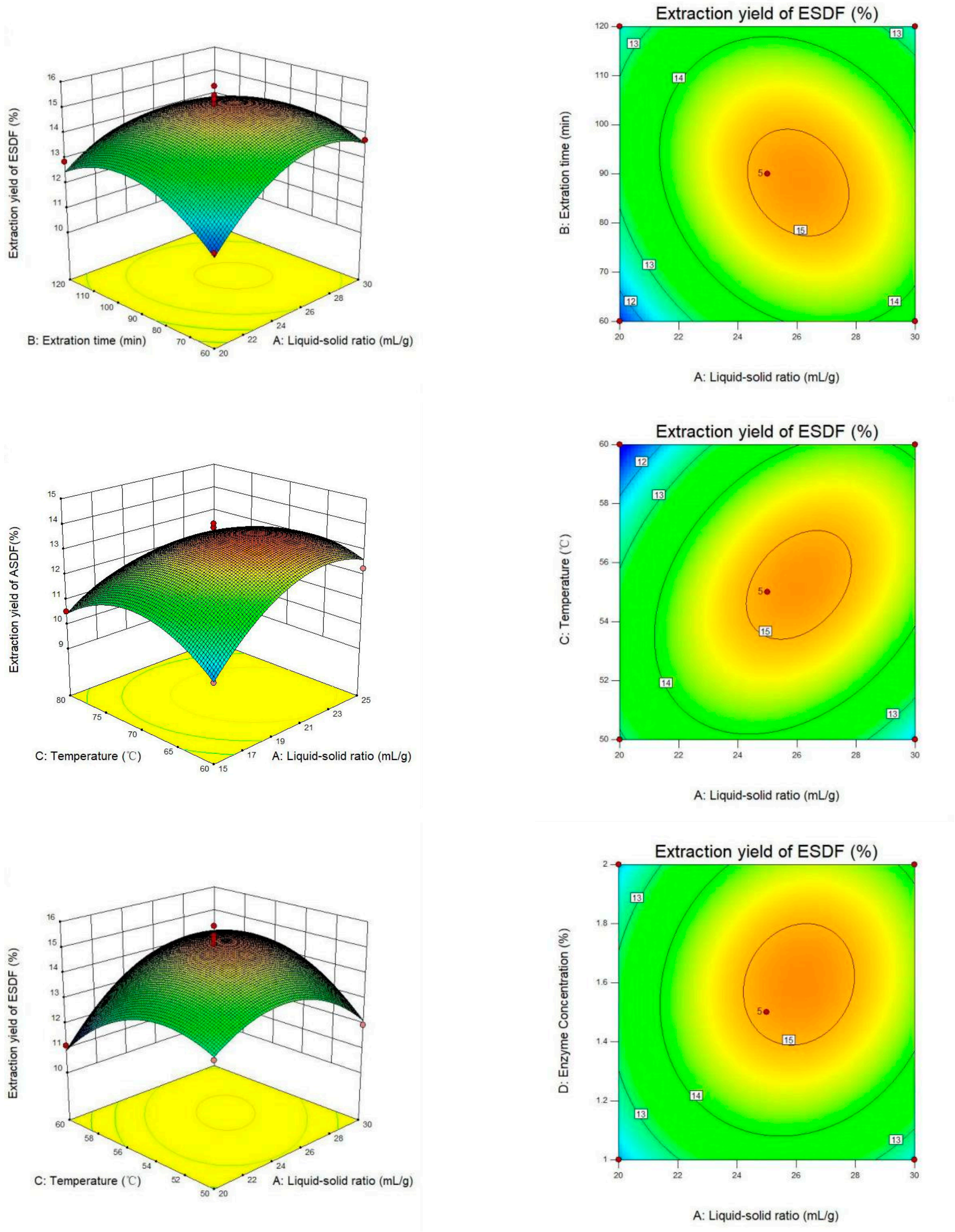

Extraction yield of ESDF (%)
B: Extration time (min)
A: Liquid-solid ratio (mL/g)
Extraction yield of ESDF (%)
B: Extration time (min)
A: Liquid-solid ratio (mL/g)
Extraction yield of ASDF(%)
C: Temperature (℃)
A: Liquid-solid ratio (mL/g)
Extraction yield of ESDF (%)
C: Temperature (℃)
A: Liquid-solid ratio (mL/g)
Extraction yield of ESDF (%)
C: Temperature (℃)
A: Liquid-solid ratio (mL/g)
Extraction yield of ESDF (%)
D: Enzyme Concentration (%)
A: Liquid-solid ratio (mL/g)

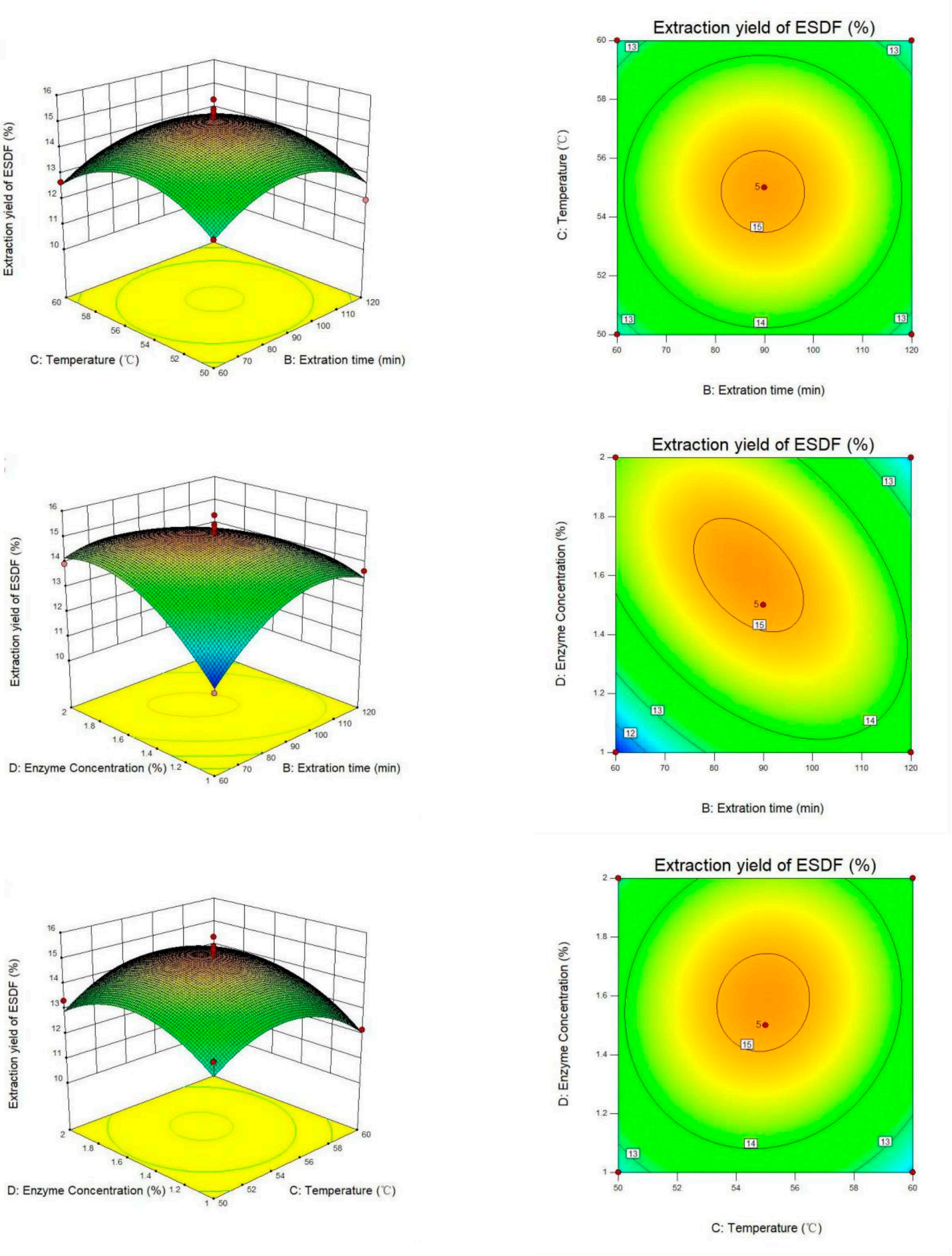
Extraction yield of ESDF (%)
C: Temperature (℃)
B: Extration time (min)
Extraction yield of ESDF (%)
C: Temperature (℃)
B: Extration time (min)
Extraction yield of ESDF (%)
D: Enzyme Concentration (%)
B: Extration time (min)
Extraction yield of ESDF (%)
D: Enzyme Concentration (%)
B: Extration time (min)
Extraction yield of ESDF (%)
D: Enzyme Concentration (%)
C: Temperature (℃)
Extraction yield of ESDF (%)
D: Enzyme Concentration (%)
C: Temperature (℃)
