# Supplementary material for: Extraction of Soluble Dietary Fiber from Sunflower Receptacles (Helianthus annuus L.) and Its Alleviating Effect on Constipation in Mice
Source: Nutrients. 2024 Oct 26;16(21):3650. doi: 10.3390/nu16213650 (PMC11547490; doi:10.3390/nu16213650)
Supplement: Supplementary file 1 [file nutrients-16-03650-s001.zip › Tables S1-S3 (New).pdf]

Table S1. Four-factor, three-level Box-Behnken design and experimental data of ASDF.

Runs	A Liquid-solid ratio (mL/g)	B Extration time （min）	C Temperature (℃)	D Acid Concentration (%)	ASDF yield (%)
1	20	90	80	1.5	11.16
2	25	60	70	1.0	12.28
3	20	90	80	0.5	10.08
4	20	60	80	1.0	10.44
5	20	120	70	0.5	10.74
6	20	120	60	1.0	12.09
7	15	90	70	1.5	10.9
8	20	90	60	1.5	11.53
9	15	60	70	1.0	10.89
10	25	120	70	1.0	12.71
11	20	90	70	1.0	13.89
12	15	90	70	0.5	9.34
13	20	90	70	1.0	14.05
14	20	90	60	0.5	10.79
15	20	60	70	1.5	11.12
16	20	90	70	1.0	13.45
17	15	120	70	1.0	10.35
18	20	60	60	1.0	11.79
19	25	90	80	1.0	10.34
20	25	90	70	0.5	11.51
21	20	60	70	0.5	10.15
22	20	90	70	1.0	12.83
23	15	90	80	1.0	10.54
24	20	120	80	1.0	10.78
25	15	90	60	1.0	10.09
26	20	90	70	1.0	13.32
27	25	90	70	1.5	10.91
28	20	120	70	1.5	11.97
29	25	90	60	1.0	12.28

Table S2. Three-factor, three-level Box-Behnken design and experimental data of WSDF.

Runs	A<br>Liquid-solid ratio<br>(mL/g)	B<br>Extration time<br>(min)	C<br>Temperature<br>(℃)	WSDF yield<br>(%)
1	40	120	70	10.19
2	35	90	70	9.91
3	30	120	90	11.62
4	35	150	90	10.18
5	35	120	80	13.15
6	35	150	70	9.99
7	35	120	80	12.89
8	35	90	90	10.88
9	35	120	80	13.34
10	30	150	80	11.18
11	40	120	90	11.53
12	30	120	70	11.10
13	30	90	80	10.93
14	35	120	80	13.03
15	40	90	80	10.84
16	35	120	80	13.04
17	40	150	80	10.78

Table S3. Four-factor, three-level Box-Behnken design and experimental data of ESDF.

Runs	A<br>Liquid-solid ratio (mL/g)	B<br>Extration time (min)	C<br>Temperature (℃)	D<br>Enzyme Concentration (%)	ESDF yield (%)
1	25	90	55	1.5	15.84
2	30	90	60	1.5	13.98
3	25	90	50	2.0	13.35
4	25	120	55	1.0	13.66
5	25	120	55	2.0	12.18
6	25	120	50	1.5	11.98
7	20	90	50	1.5	12.78
8	30	120	55	1.5	12.83
9	25	90	55	1.5	15.48
10	30	60	55	1.5	13.75
11	25	90	50	1.0	13.09
12	30	90	55	2.0	13.65
13	25	60	50	1.5	12.67
14	20	90	55	1.0	11.61
15	25	120	60	1.5	11.88
16	25	60	60	1.5	12.68
17	25	90	60	2.0	13.15
18	25	90	60	1.0	12.19
19	25	90	55	1.5	13.68
20	30	90	50	1.5	11.96
21	20	60	55	1.5	11.59
22	25	90	55	1.5	15.15
23	25	90	55	1.5	15.34
24	20	90	60	1.5	11.12
25	25	60	55	2.0	13.94
26	25	60	55	1.0	11.15
27	30	90	55	1.0	12.09
28	20	90	55	2.0	11.84
29	20	120	55	1.5	12.89
