# Supplementary figures and images for: Extraction of Soluble Dietary Fiber from Sunflower Receptacles (Helianthus annuus L.) and Its Alleviating Effect on Constipation in Mice
Source: Nutrients. 2024 Oct 26;16(21):3650. doi: 10.3390/nu16213650 (PMC11547490; doi:10.3390/nu16213650)

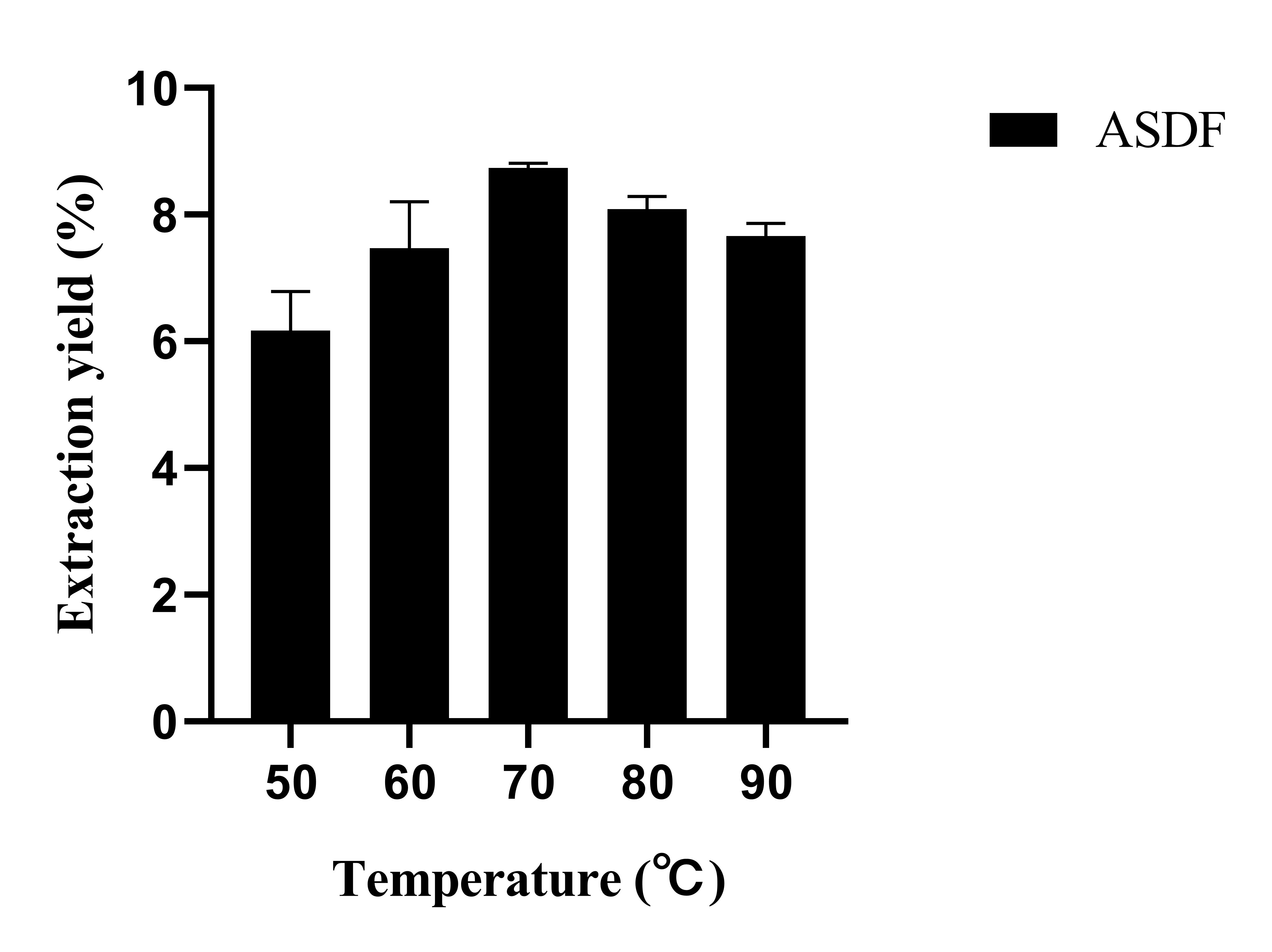

Supplement: Supplementary file 1 [file nutrients-16-03650-s001.zip › Fig. S1-3 Single factor experimental results/S1-A.jpg]

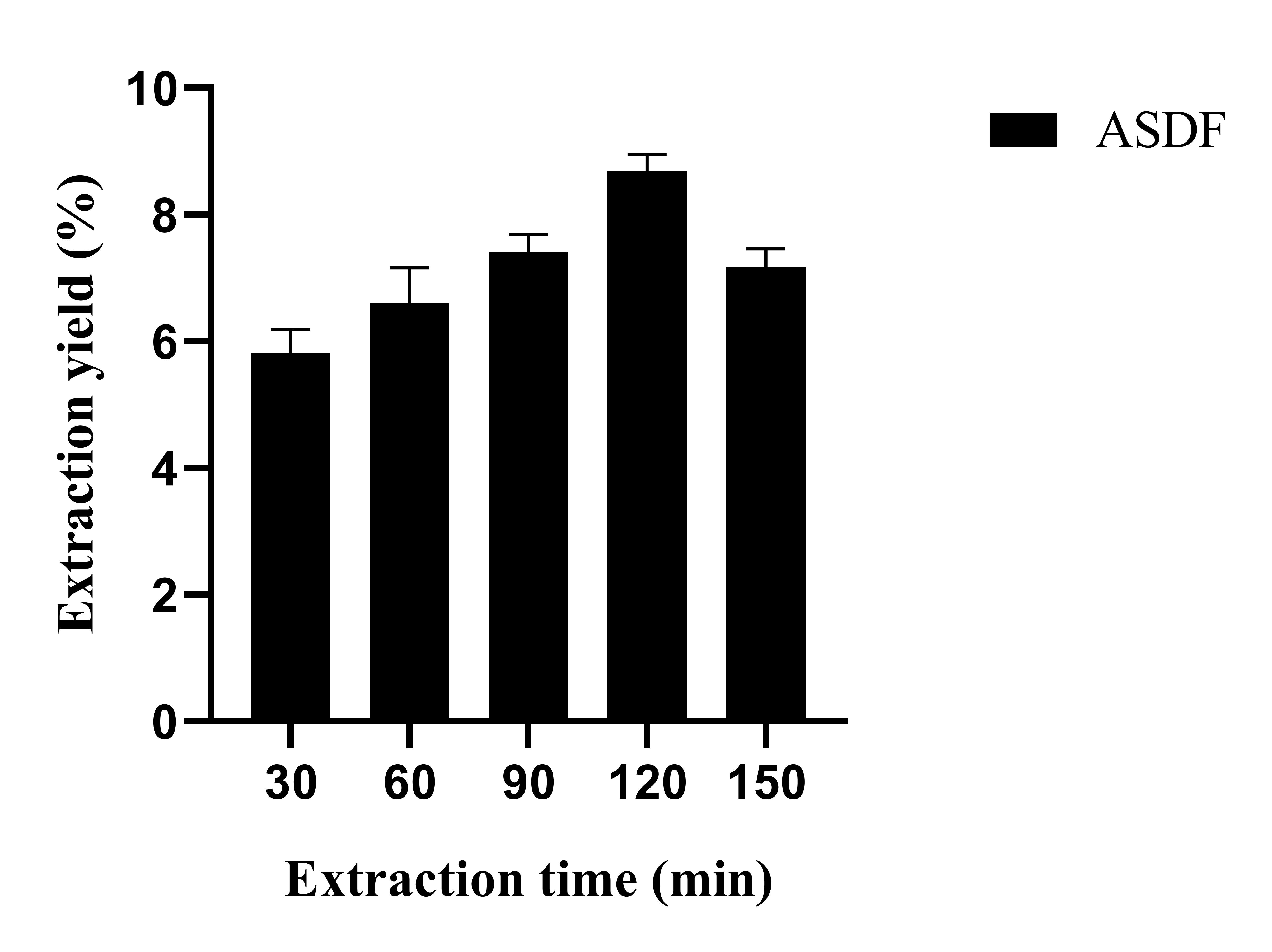

Supplement: Supplementary file 1 [file nutrients-16-03650-s001.zip › Fig. S1-3 Single factor experimental results/S1-B.jpg]

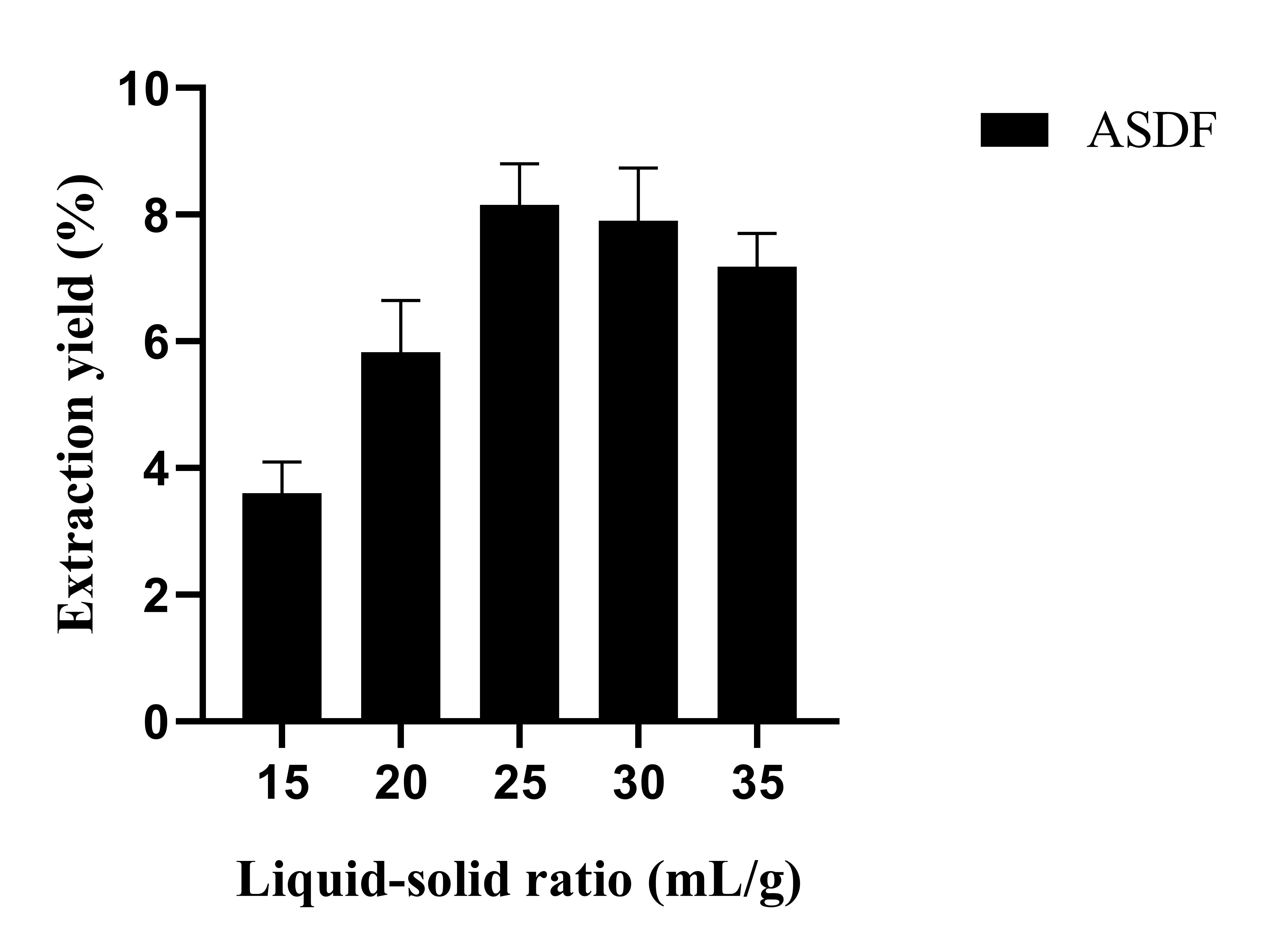

Supplement: Supplementary file 1 [file nutrients-16-03650-s001.zip › Fig. S1-3 Single factor experimental results/S1-C.jpg]

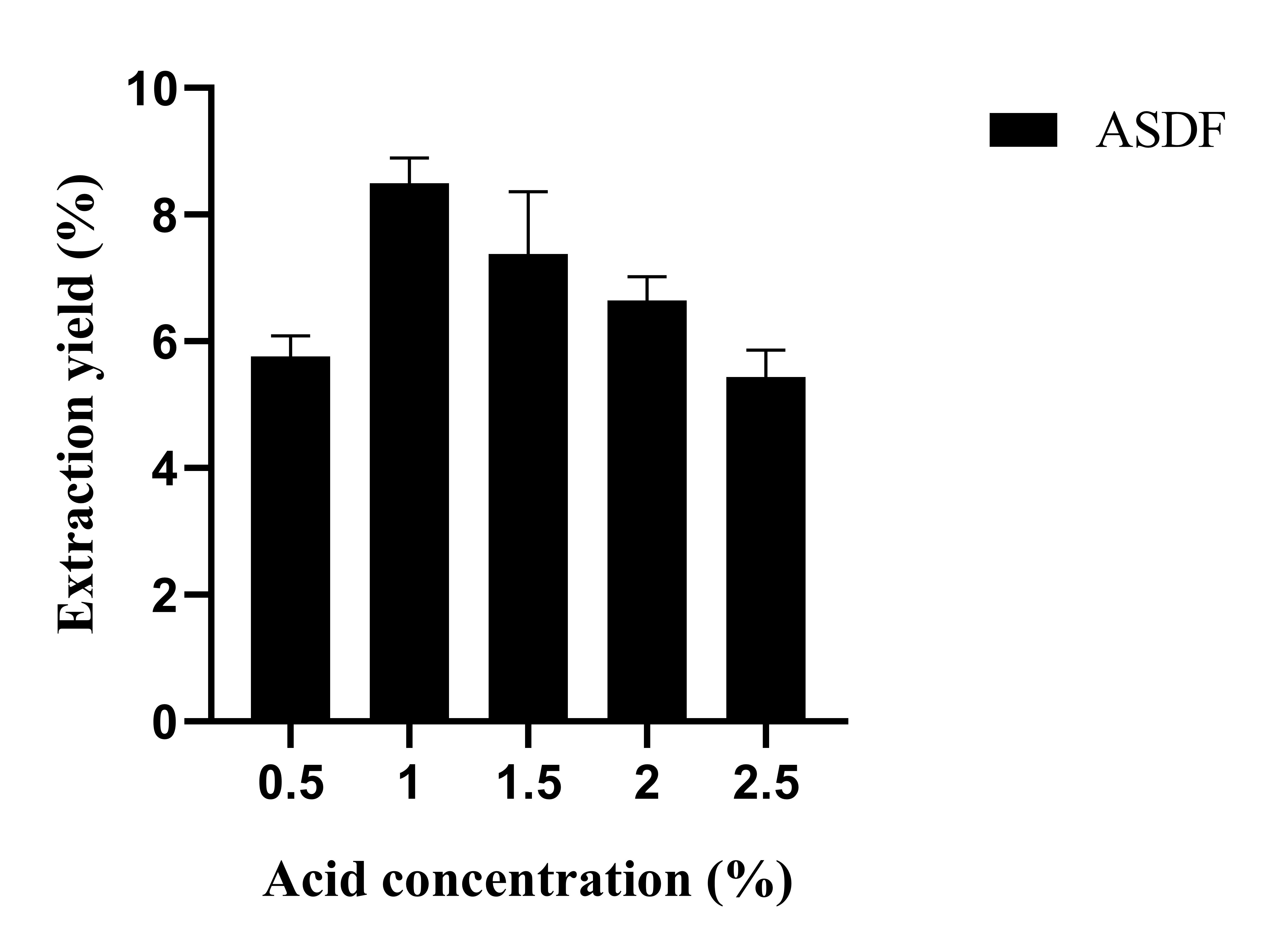

Supplement: Supplementary file 1 [file nutrients-16-03650-s001.zip › Fig. S1-3 Single factor experimental results/S1-D.jpg]

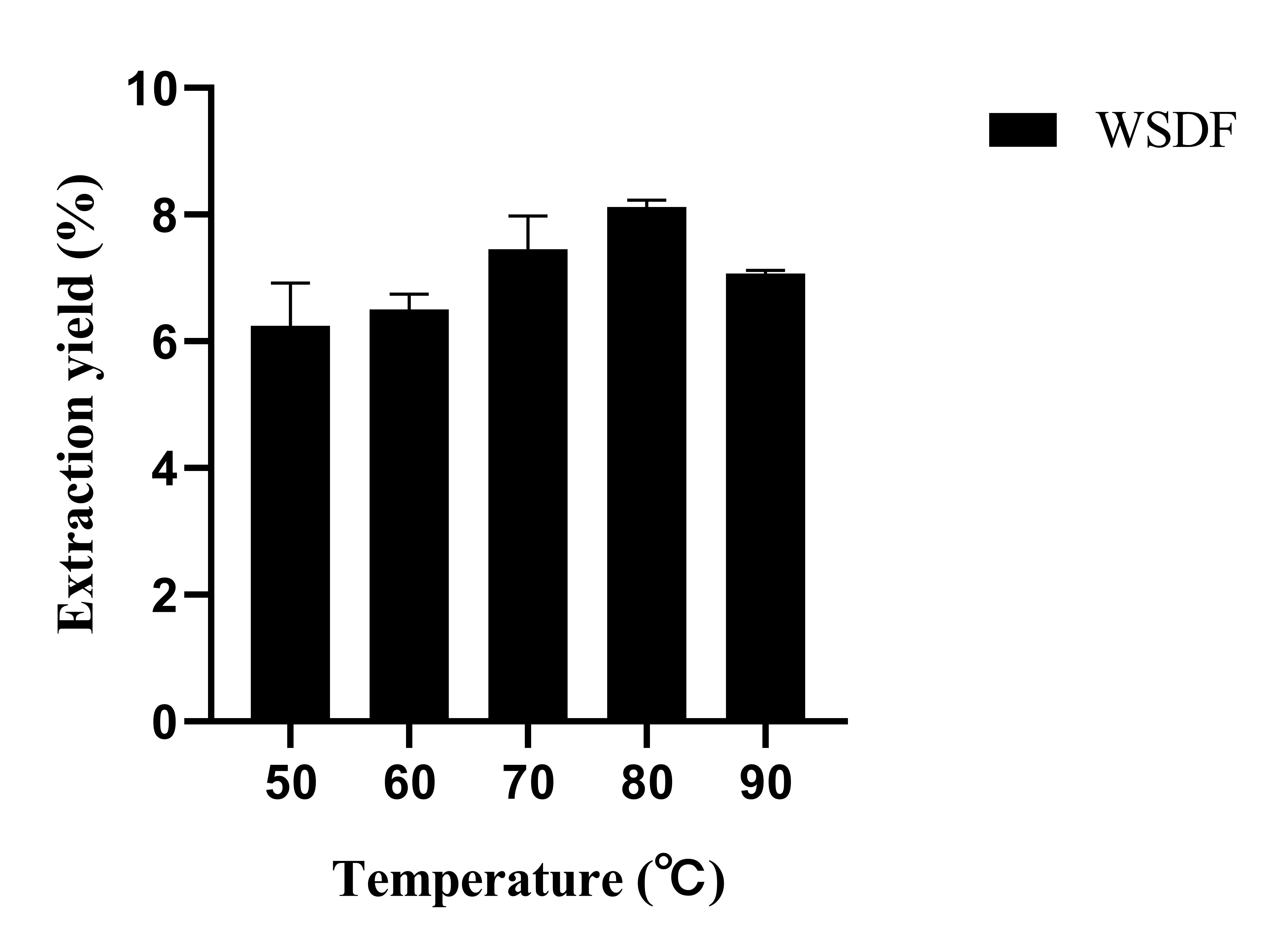

Supplement: Supplementary file 1 [file nutrients-16-03650-s001.zip › Fig. S1-3 Single factor experimental results/S2-A.jpg]

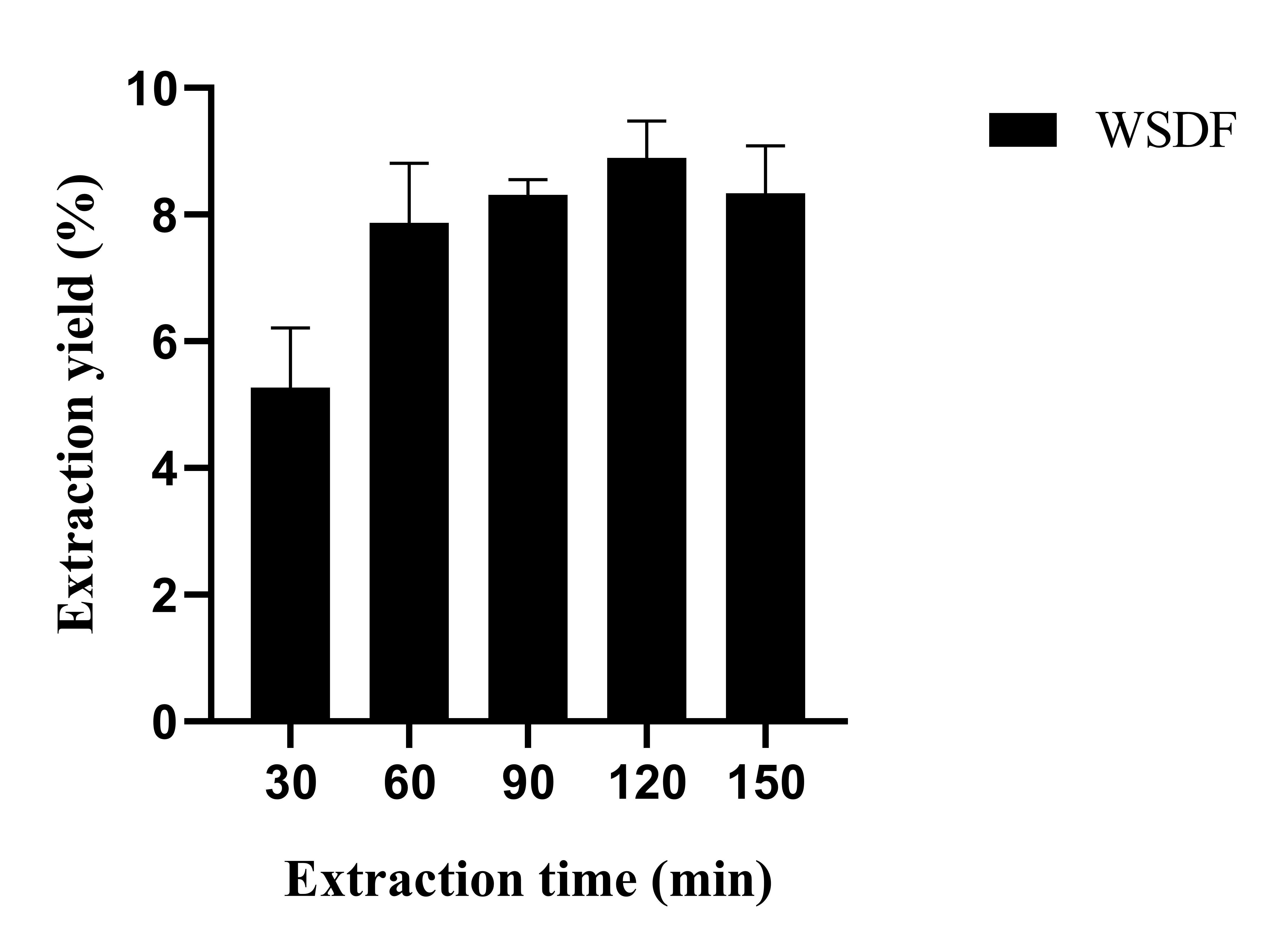

Supplement: Supplementary file 1 [file nutrients-16-03650-s001.zip › Fig. S1-3 Single factor experimental results/S2-B.jpg]

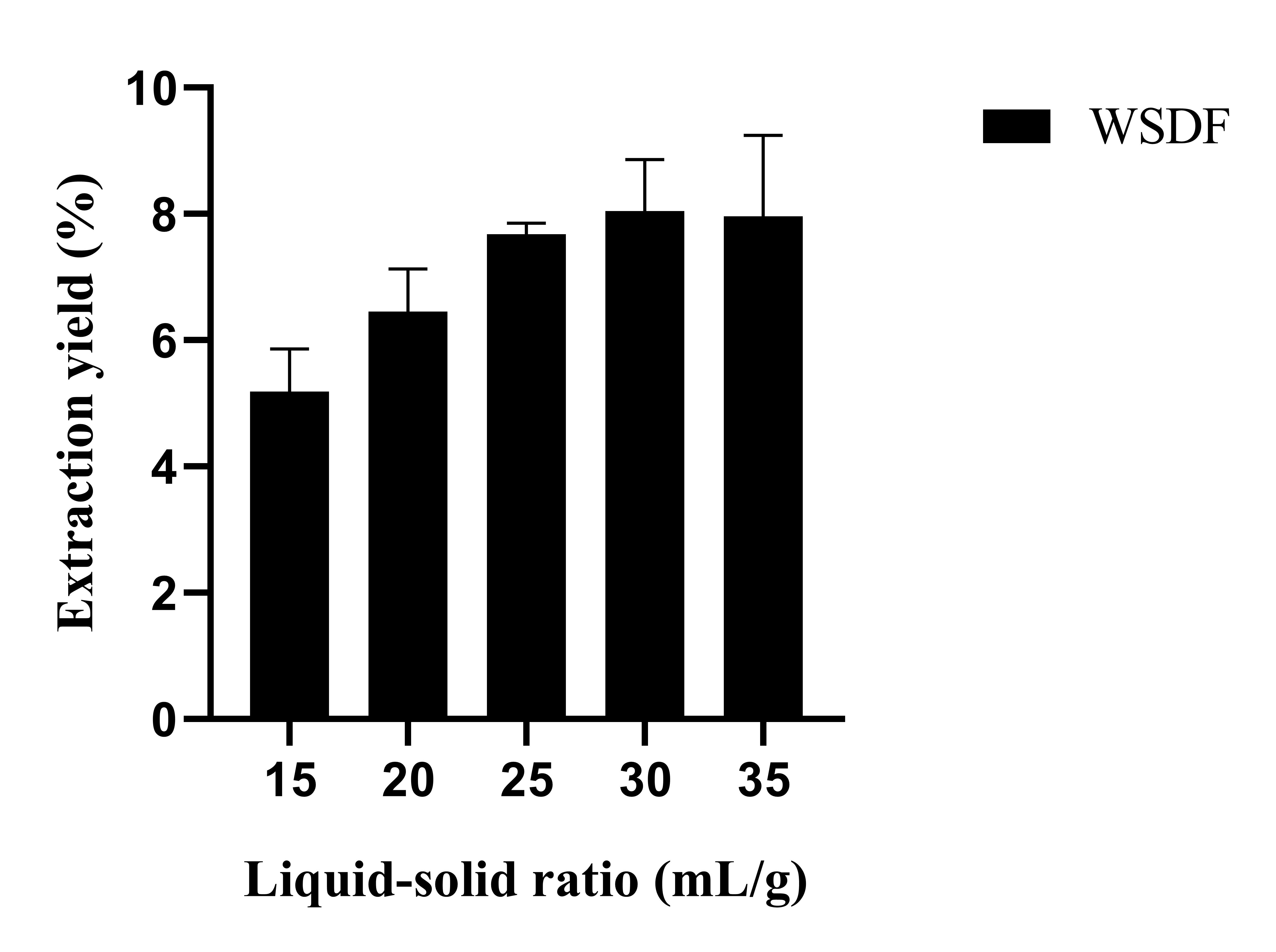

Supplement: Supplementary file 1 [file nutrients-16-03650-s001.zip › Fig. S1-3 Single factor experimental results/S2-C.jpg]

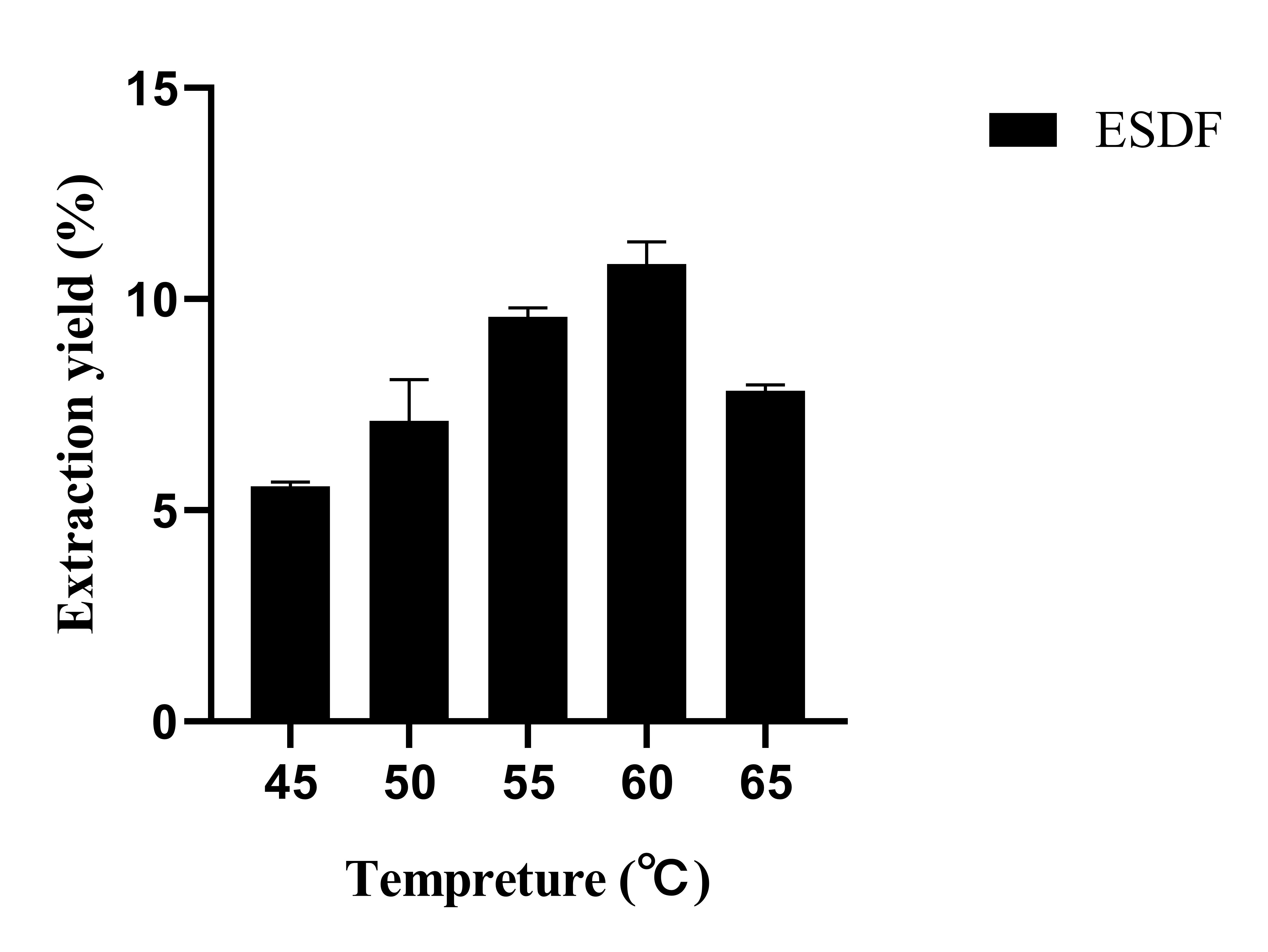

Supplement: Supplementary file 1 [file nutrients-16-03650-s001.zip › Fig. S1-3 Single factor experimental results/S3-A.jpg]

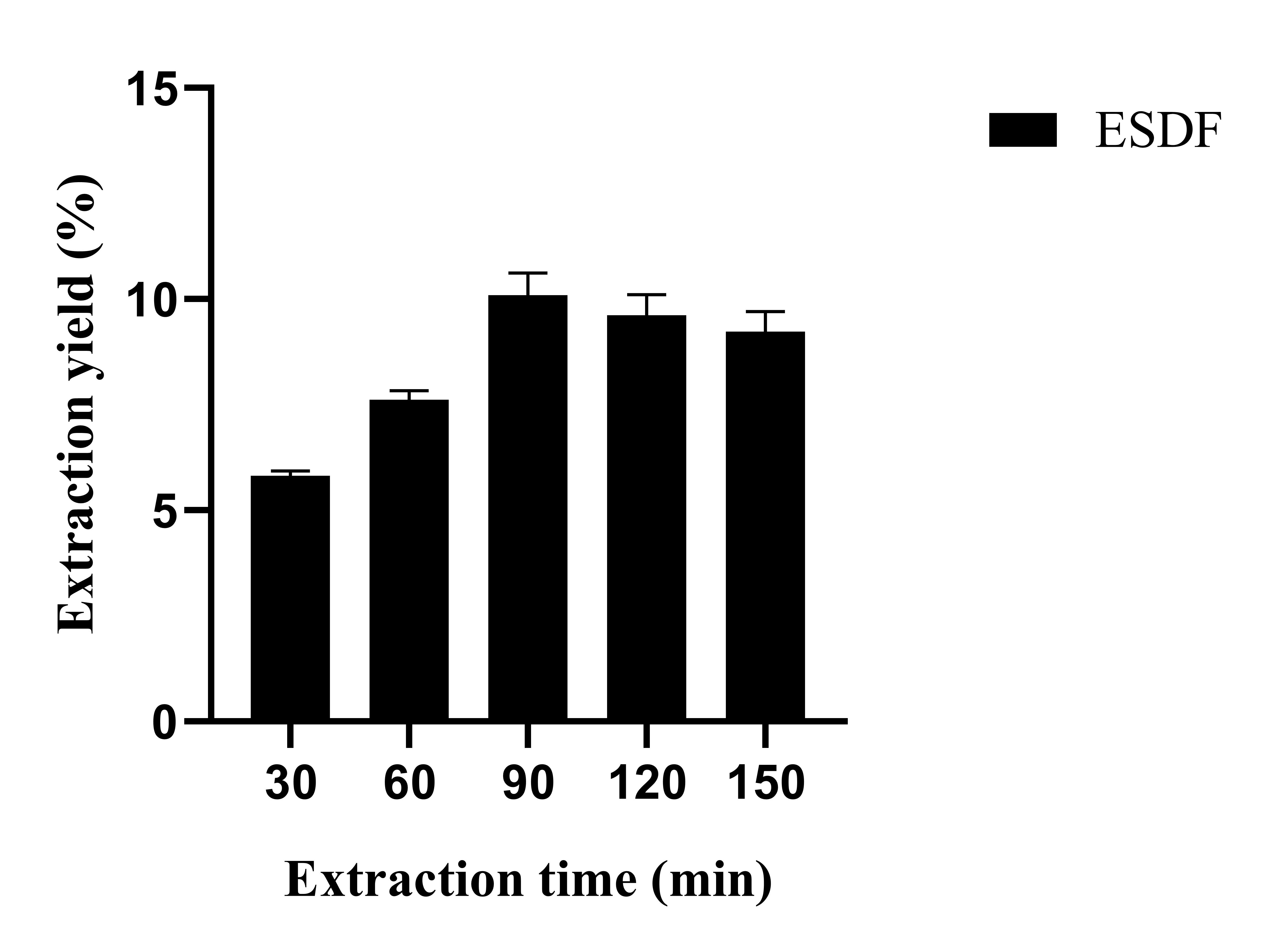

Supplement: Supplementary file 1 [file nutrients-16-03650-s001.zip › Fig. S1-3 Single factor experimental results/S3-B.jpg]

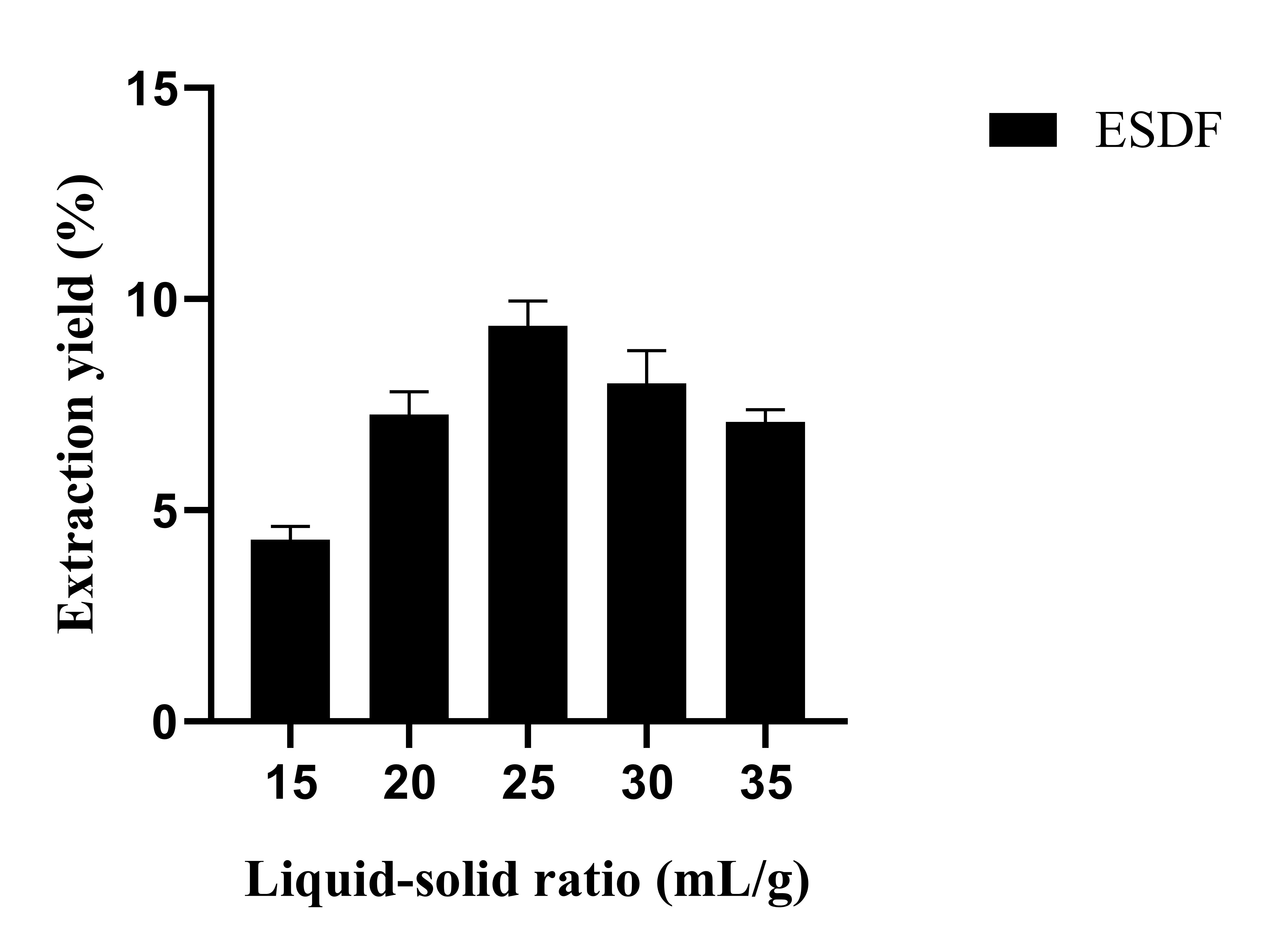

Supplement: Supplementary file 1 [file nutrients-16-03650-s001.zip › Fig. S1-3 Single factor experimental results/S3-C.jpg]

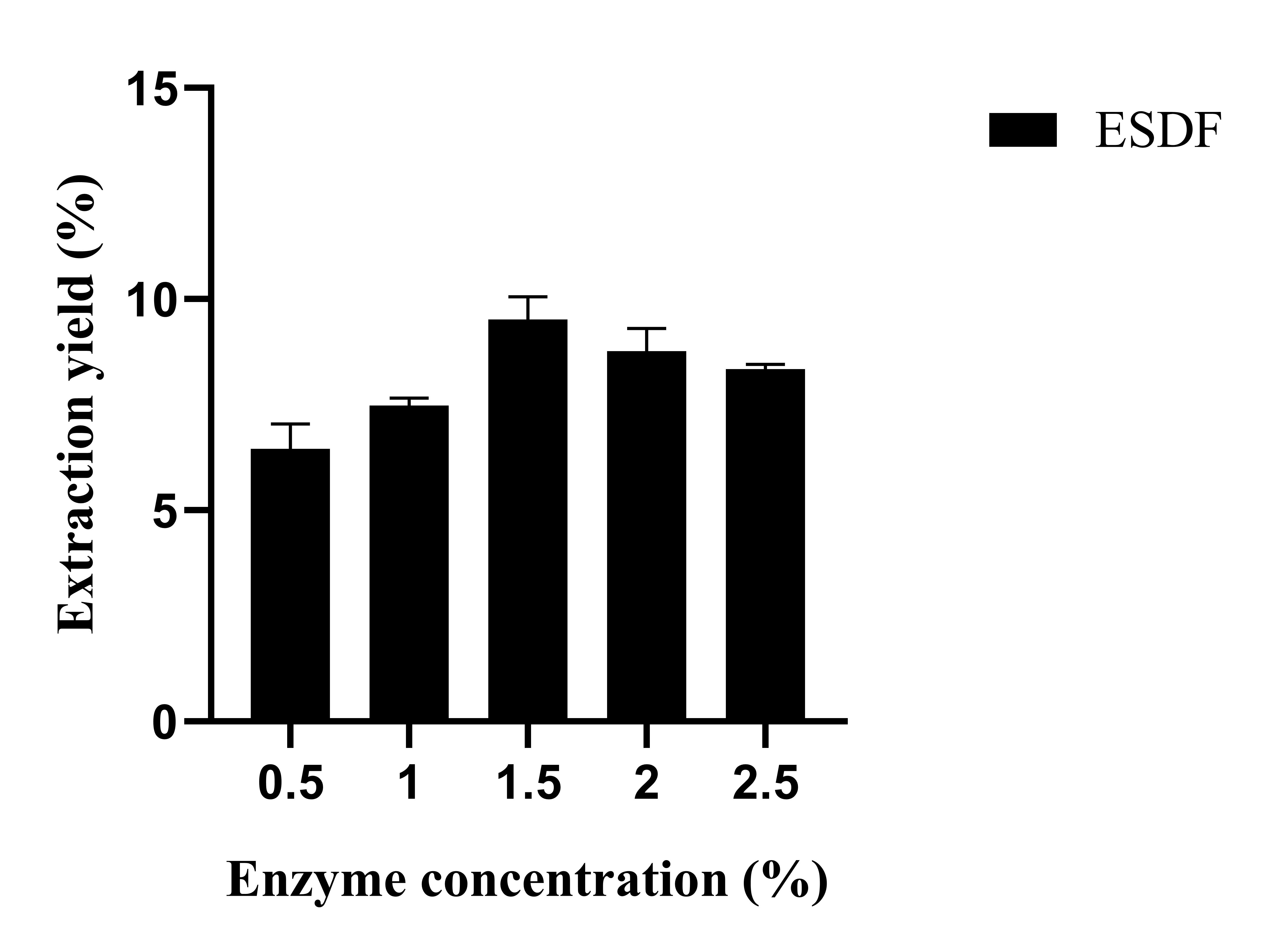

Supplement: Supplementary file 1 [file nutrients-16-03650-s001.zip › Fig. S1-3 Single factor experimental results/S3-D.jpg]

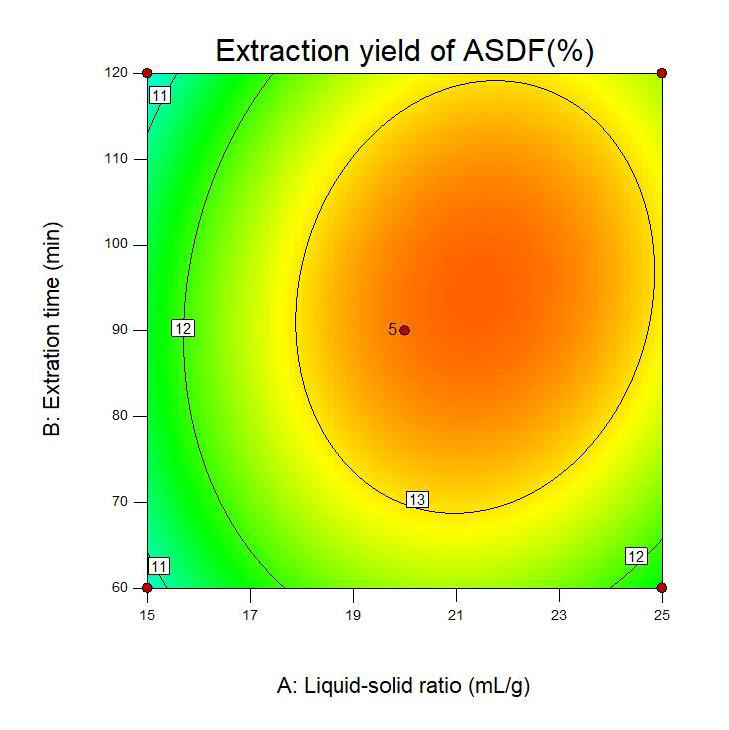

Supplement: Supplementary file 1 [file nutrients-16-03650-s001.zip › Fig. S4-6Response surface experimental results/ASDF-AB.jpg]

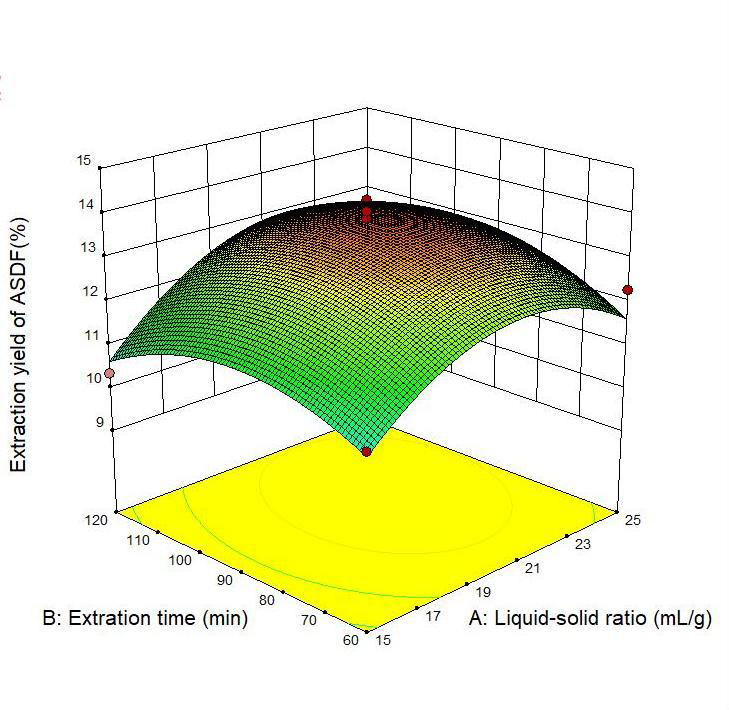

Supplement: Supplementary file 1 [file nutrients-16-03650-s001.zip › Fig. S4-6Response surface experimental results/ASDF-AB2.jpg]

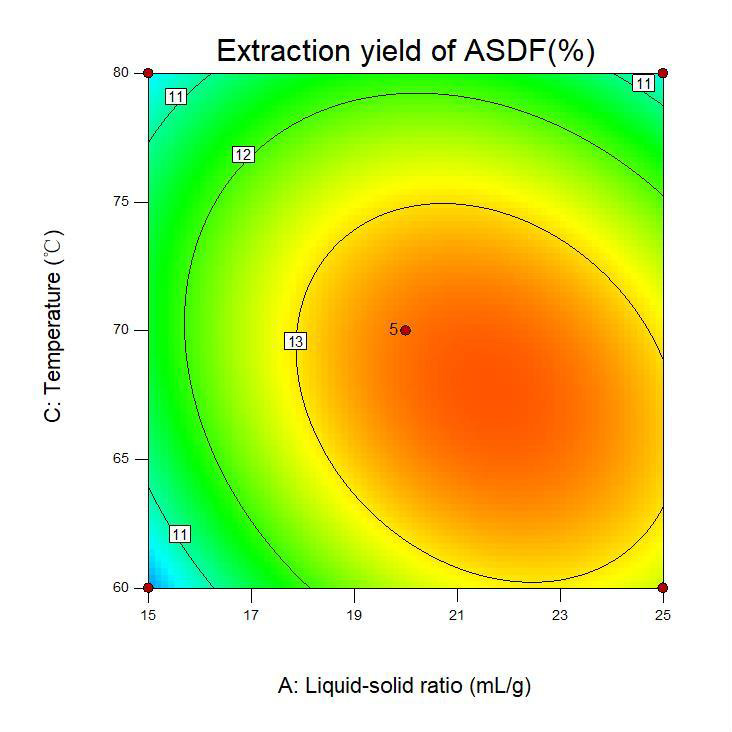

Supplement: Supplementary file 1 [file nutrients-16-03650-s001.zip › Fig. S4-6Response surface experimental results/ASDF-AC.jpg]

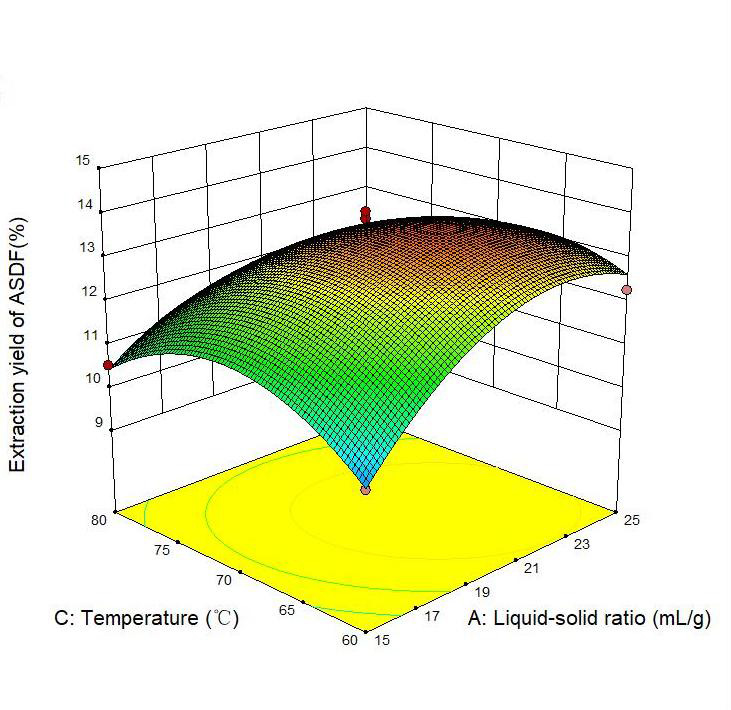

Supplement: Supplementary file 1 [file nutrients-16-03650-s001.zip › Fig. S4-6Response surface experimental results/ASDF-AC2.jpg]

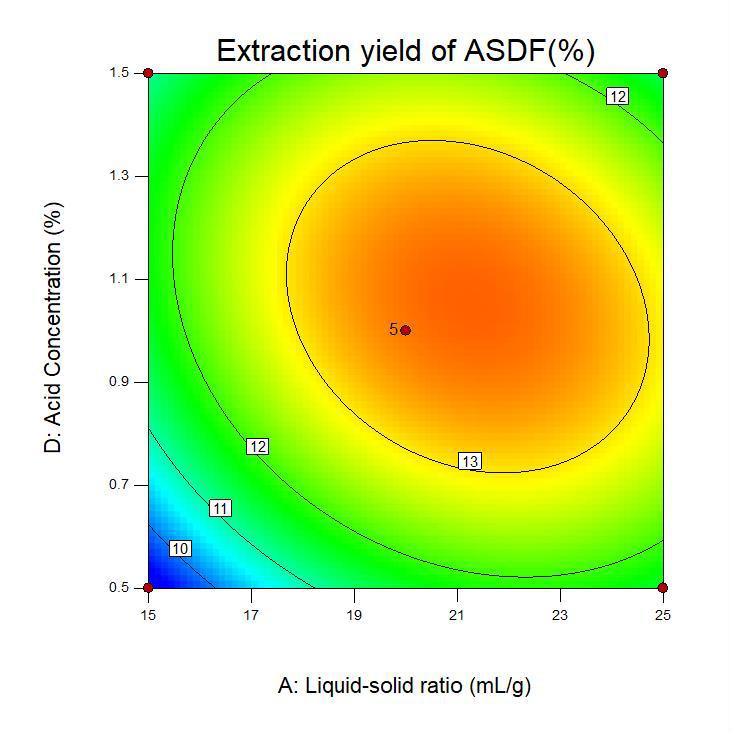

Supplement: Supplementary file 1 [file nutrients-16-03650-s001.zip › Fig. S4-6Response surface experimental results/ASDF-AD.jpg]

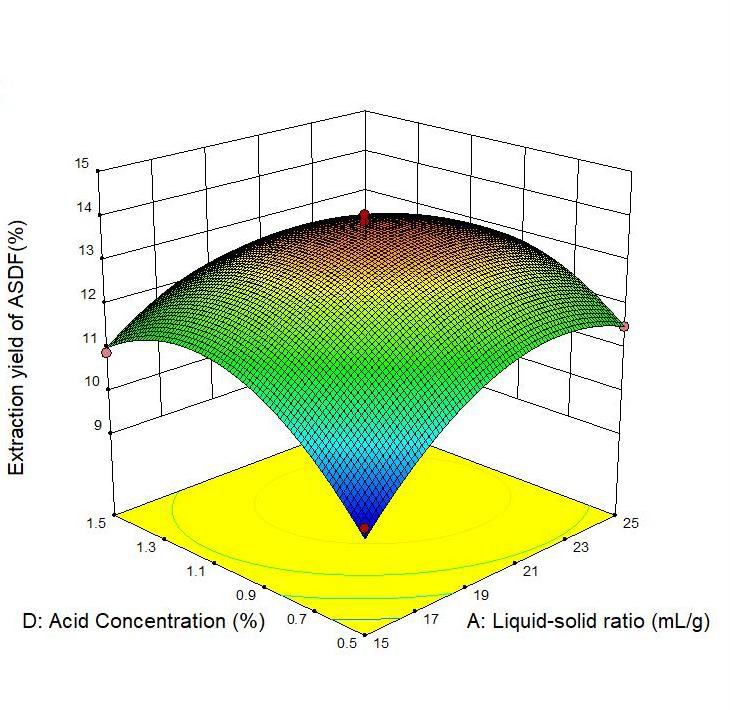

Supplement: Supplementary file 1 [file nutrients-16-03650-s001.zip › Fig. S4-6Response surface experimental results/ASDF-AD2.jpg]

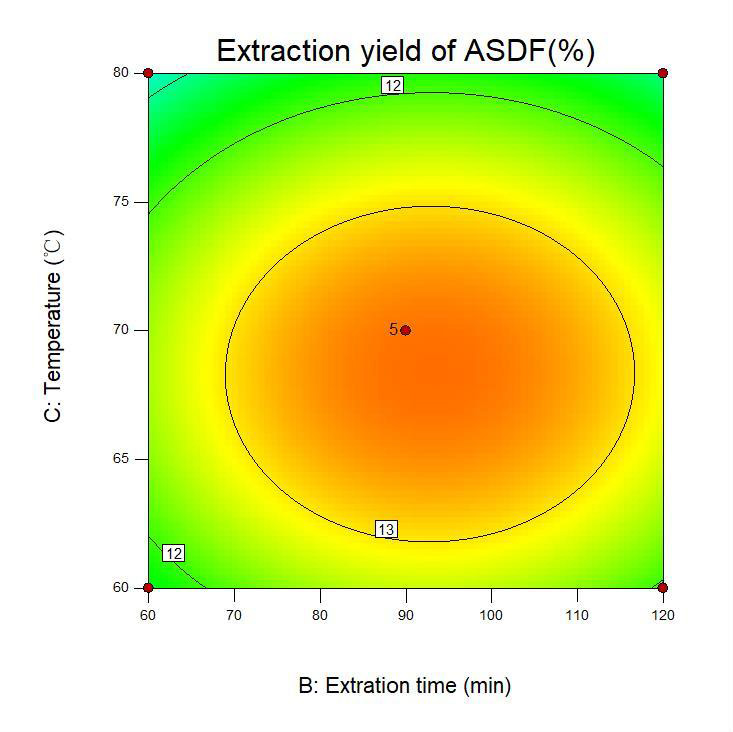

Supplement: Supplementary file 1 [file nutrients-16-03650-s001.zip › Fig. S4-6Response surface experimental results/ASDF-BC.jpg]

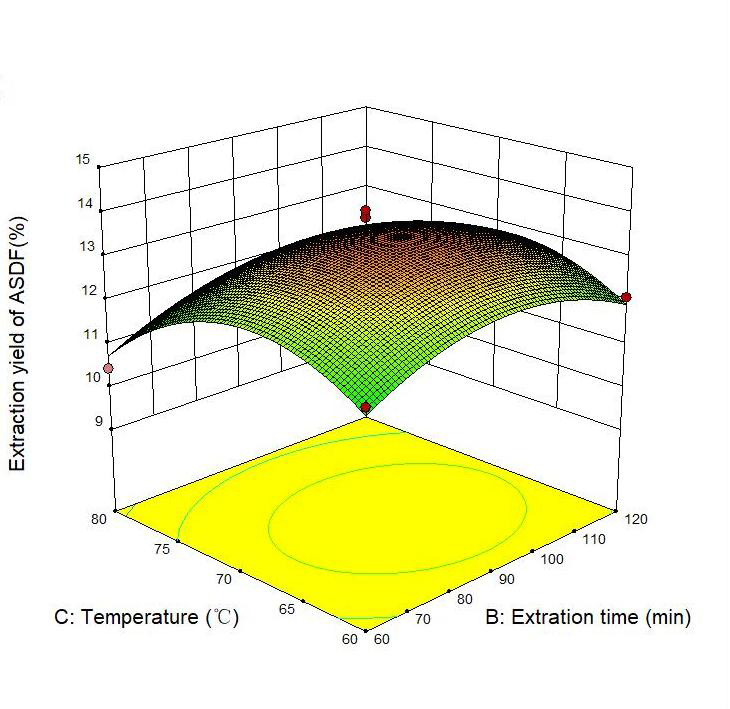

Supplement: Supplementary file 1 [file nutrients-16-03650-s001.zip › Fig. S4-6Response surface experimental results/ASDF-BC2.jpg]

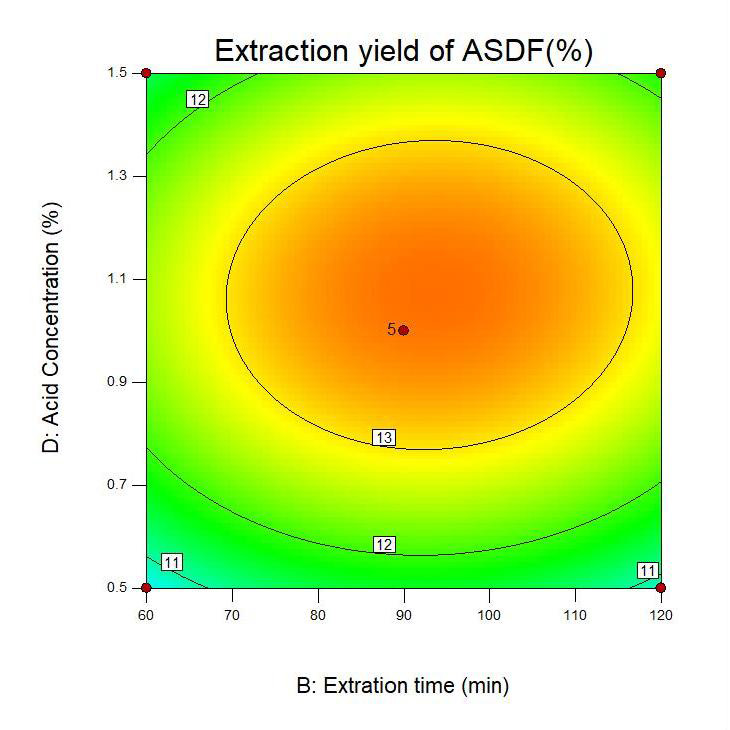

Supplement: Supplementary file 1 [file nutrients-16-03650-s001.zip › Fig. S4-6Response surface experimental results/ASDF-BD.jpg]

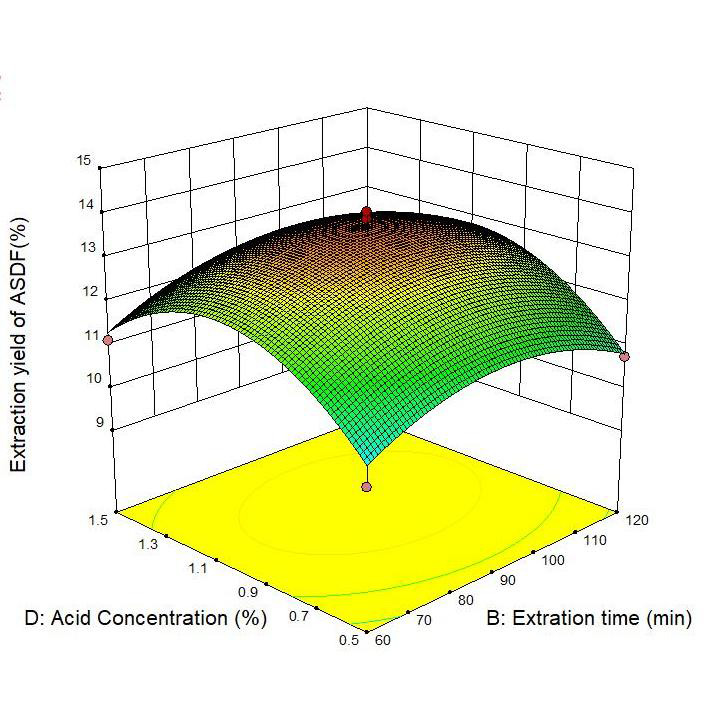

Supplement: Supplementary file 1 [file nutrients-16-03650-s001.zip › Fig. S4-6Response surface experimental results/ASDF-BD2.jpg]

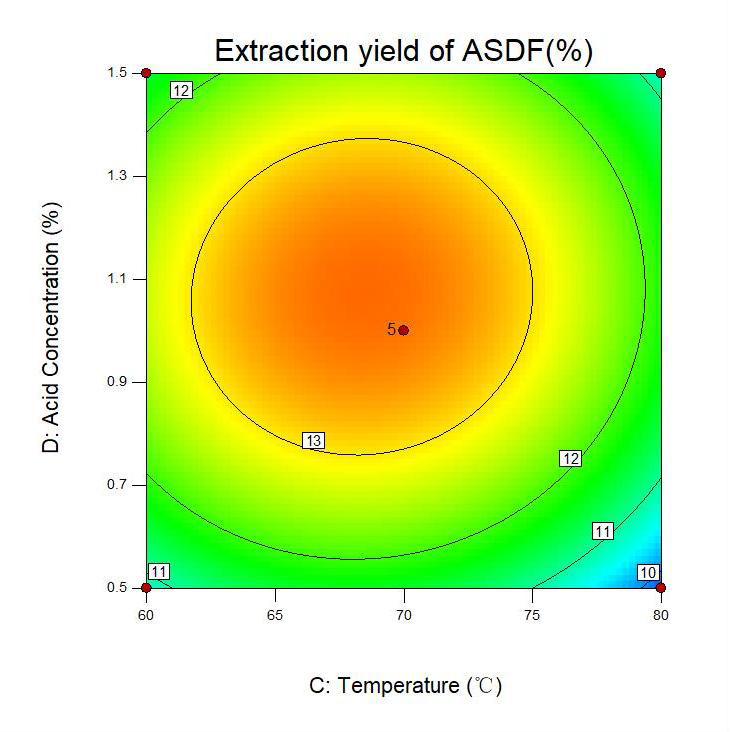

Supplement: Supplementary file 1 [file nutrients-16-03650-s001.zip › Fig. S4-6Response surface experimental results/ASDF-CD.jpg]

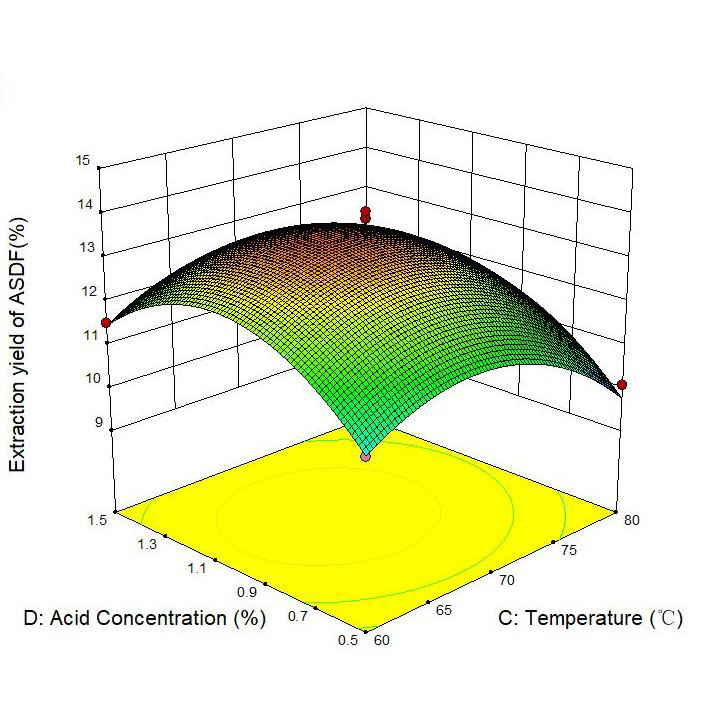

Supplement: Supplementary file 1 [file nutrients-16-03650-s001.zip › Fig. S4-6Response surface experimental results/ASDF-CD2.jpg]

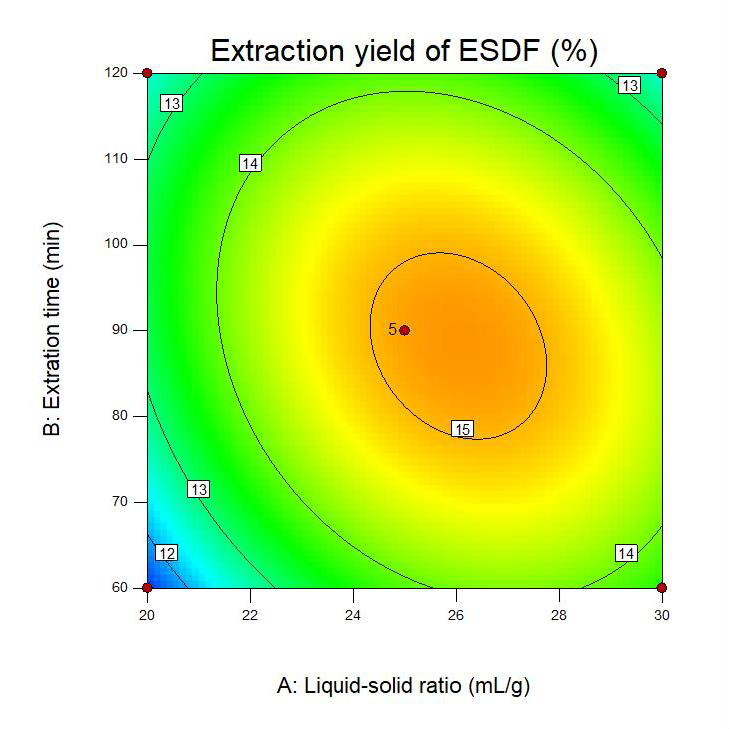

Supplement: Supplementary file 1 [file nutrients-16-03650-s001.zip › Fig. S4-6Response surface experimental results/ESDF-AB.jpg]

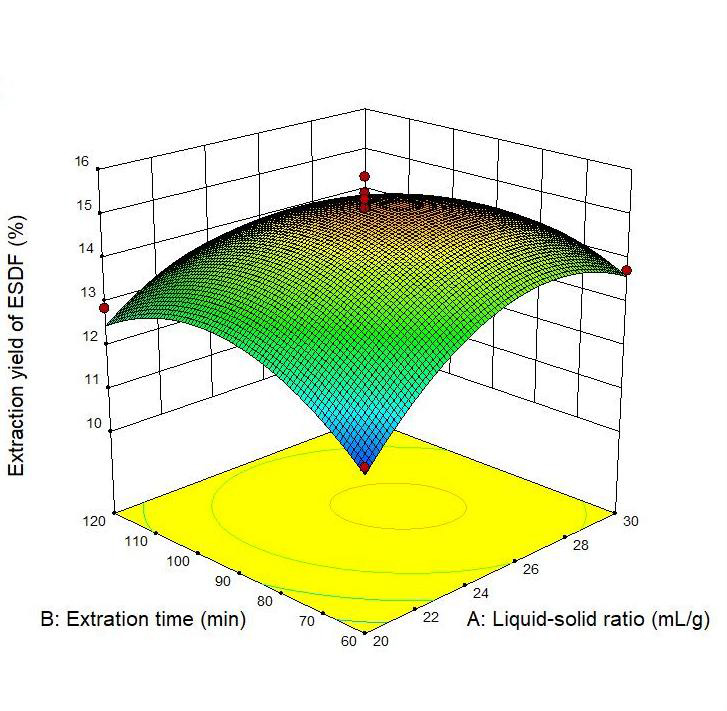

Supplement: Supplementary file 1 [file nutrients-16-03650-s001.zip › Fig. S4-6Response surface experimental results/ESDF-AB2.jpg]

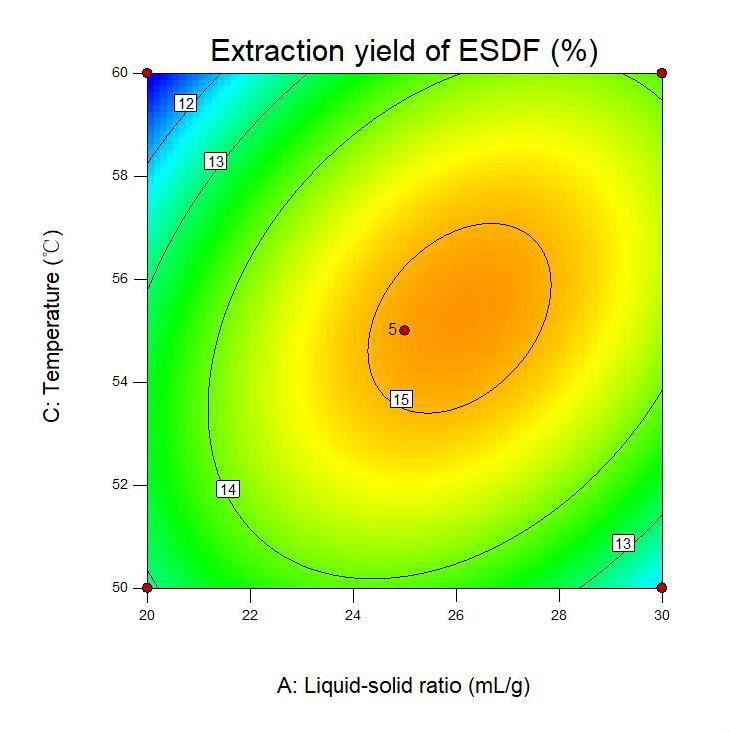

Supplement: Supplementary file 1 [file nutrients-16-03650-s001.zip › Fig. S4-6Response surface experimental results/ESDF-AC.jpg]

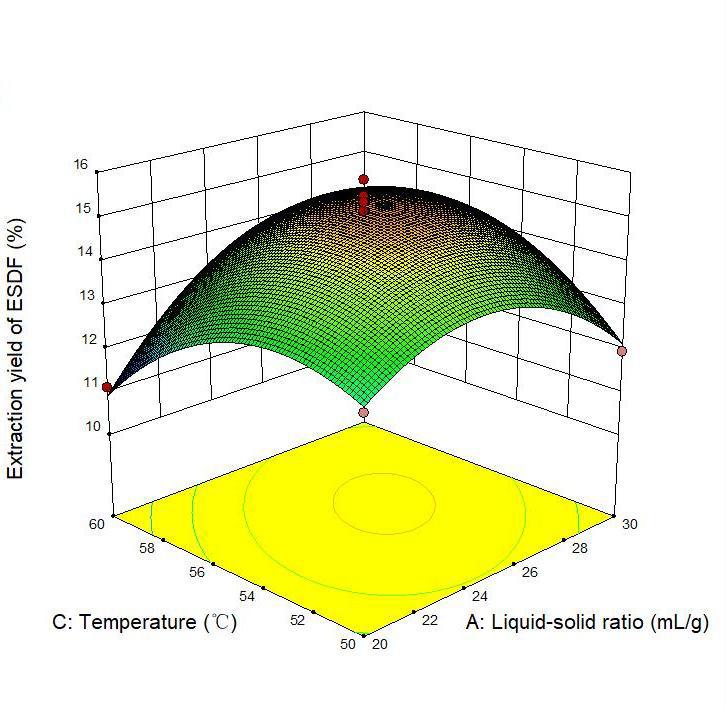

Supplement: Supplementary file 1 [file nutrients-16-03650-s001.zip › Fig. S4-6Response surface experimental results/ESDF-AC2.jpg]

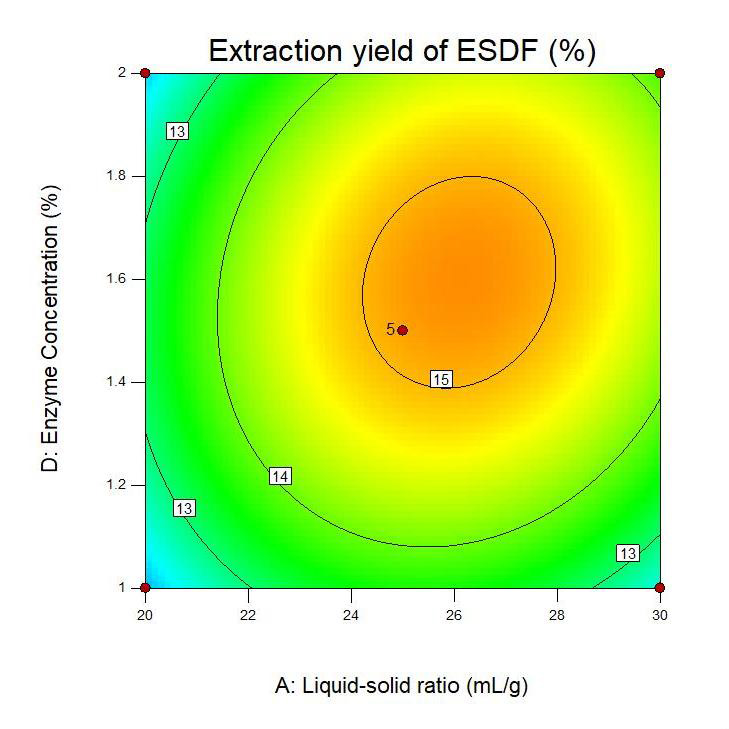

Supplement: Supplementary file 1 [file nutrients-16-03650-s001.zip › Fig. S4-6Response surface experimental results/ESDF-AD.jpg]

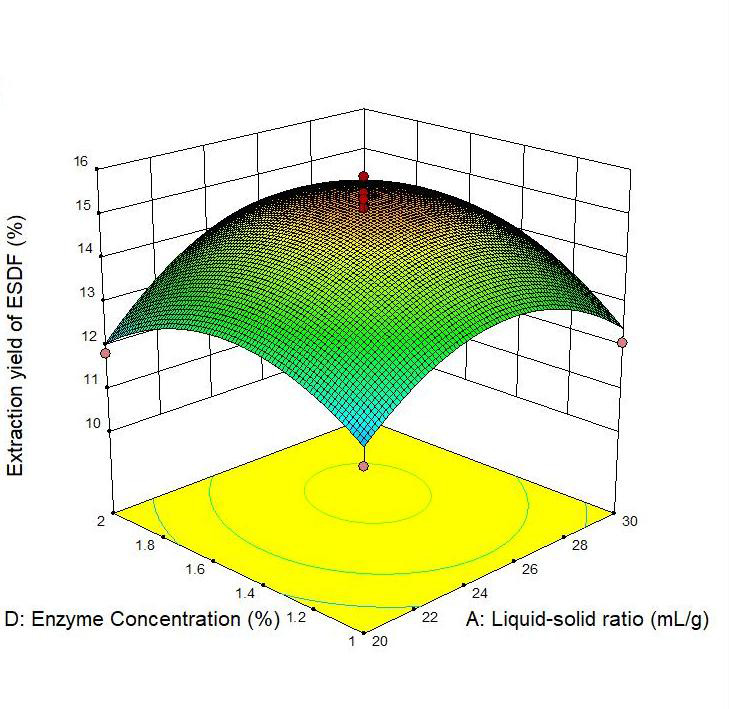

Supplement: Supplementary file 1 [file nutrients-16-03650-s001.zip › Fig. S4-6Response surface experimental results/ESDF-AD2.jpg]

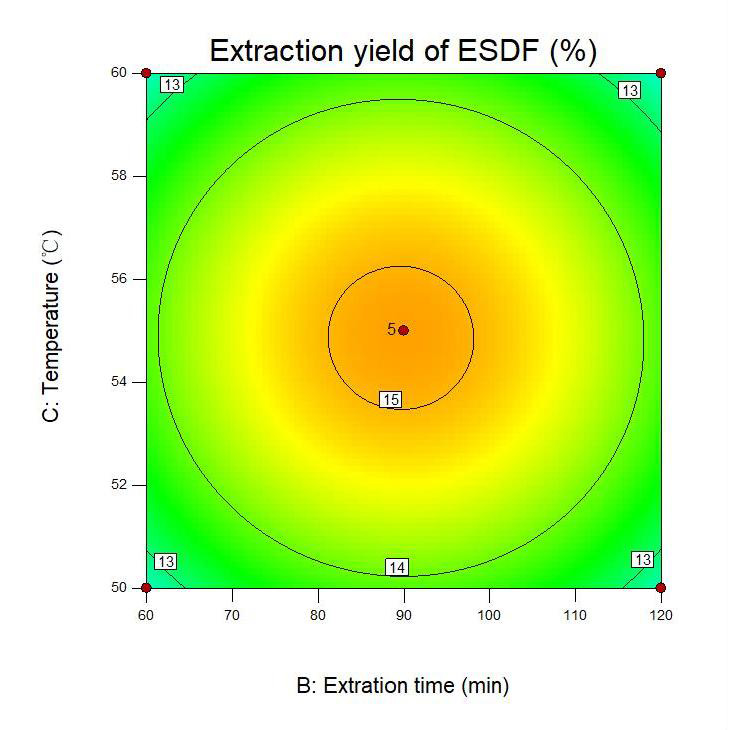

Supplement: Supplementary file 1 [file nutrients-16-03650-s001.zip › Fig. S4-6Response surface experimental results/ESDF-BC.jpg]

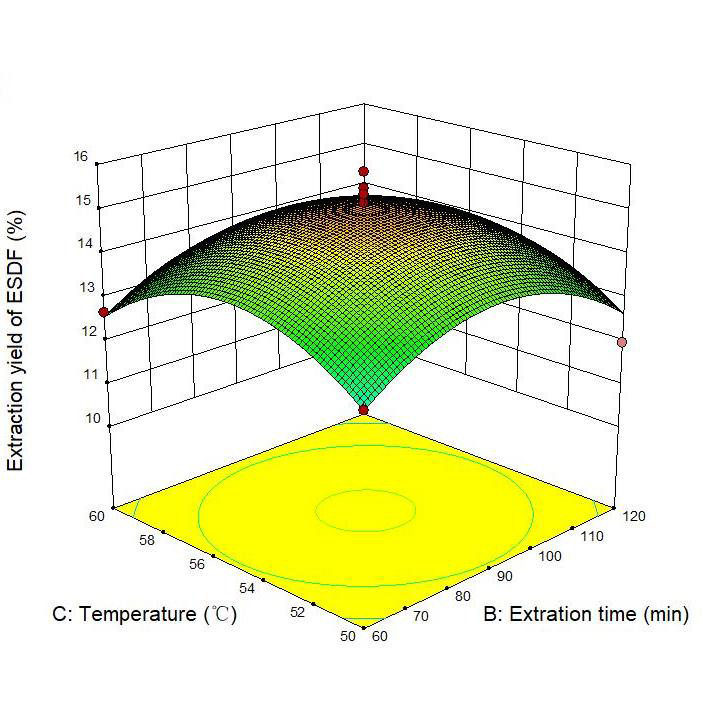

Supplement: Supplementary file 1 [file nutrients-16-03650-s001.zip › Fig. S4-6Response surface experimental results/ESDF-BC2.jpg]

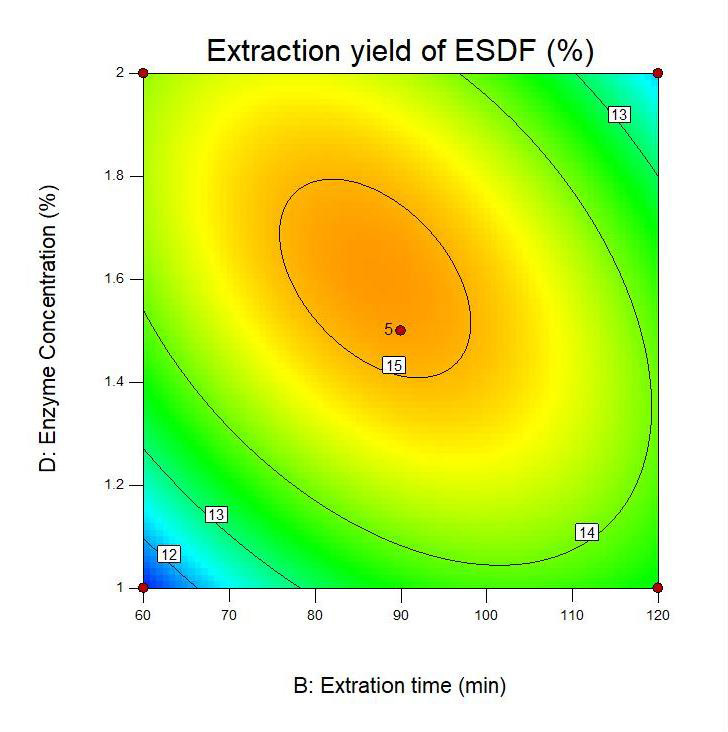

Supplement: Supplementary file 1 [file nutrients-16-03650-s001.zip › Fig. S4-6Response surface experimental results/ESDF-BD.jpg]

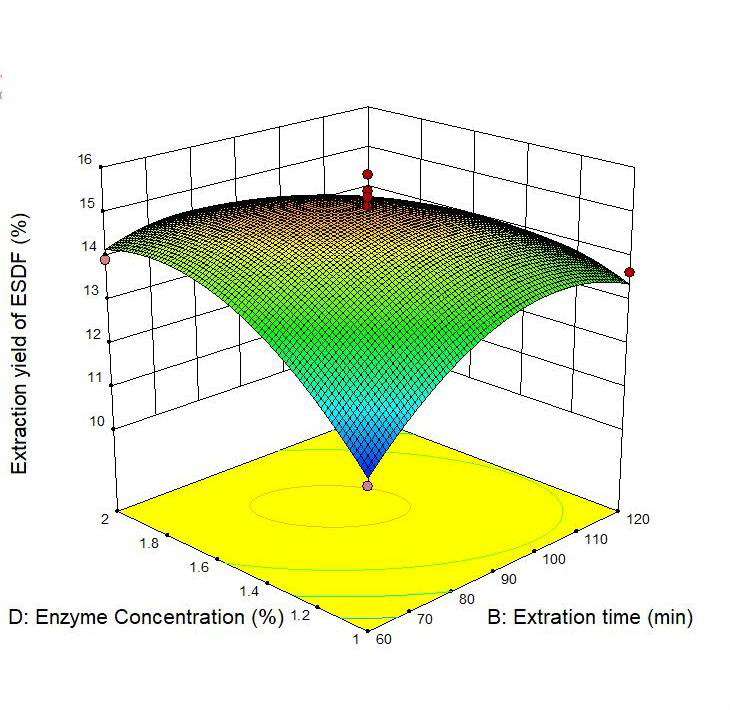

Supplement: Supplementary file 1 [file nutrients-16-03650-s001.zip › Fig. S4-6Response surface experimental results/ESDF-BD2.jpg]

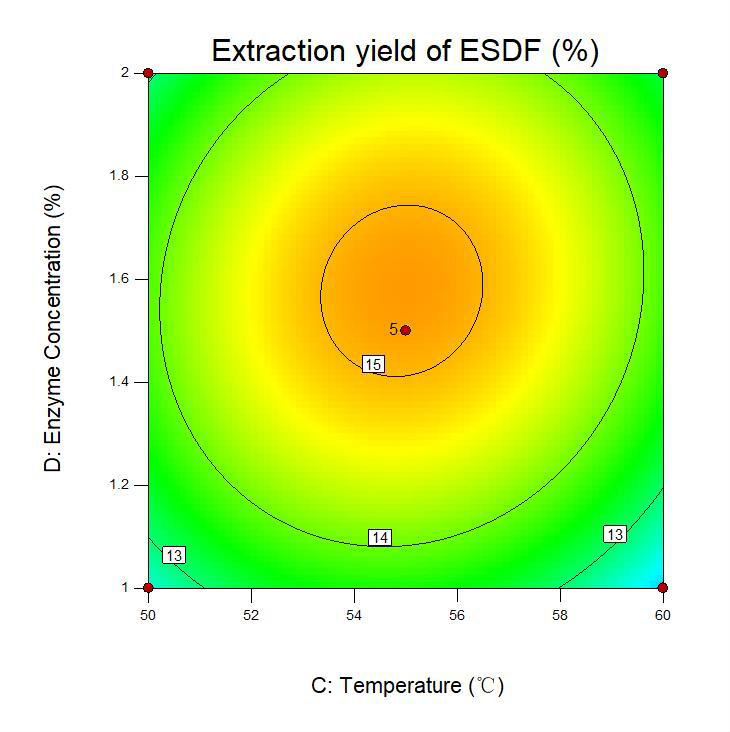

Supplement: Supplementary file 1 [file nutrients-16-03650-s001.zip › Fig. S4-6Response surface experimental results/ESDF-CD.jpg]

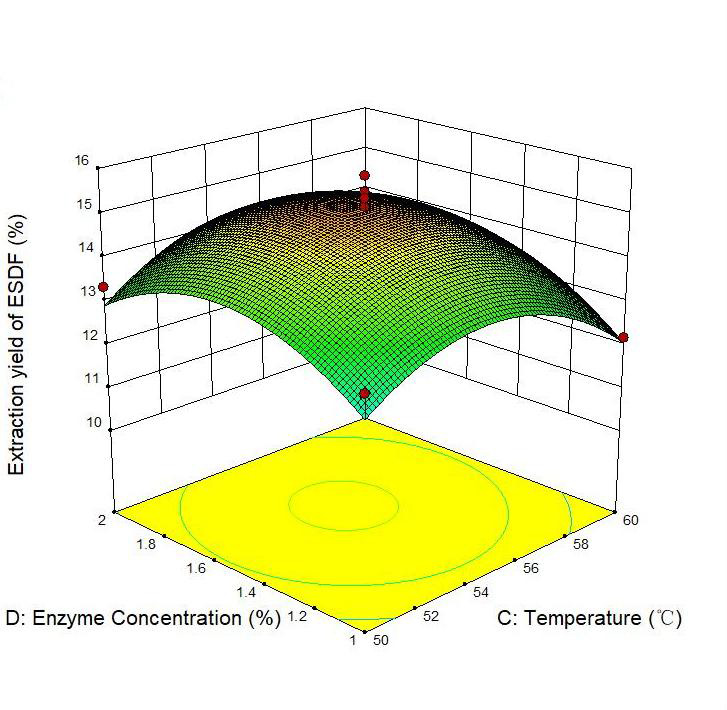

Supplement: Supplementary file 1 [file nutrients-16-03650-s001.zip › Fig. S4-6Response surface experimental results/ESDF-CD2.jpg]

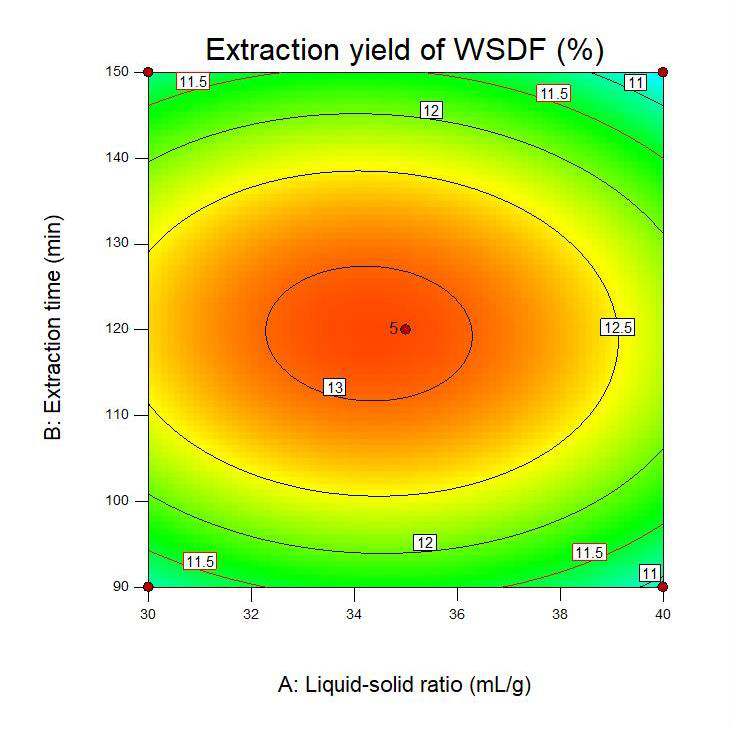

Supplement: Supplementary file 1 [file nutrients-16-03650-s001.zip › Fig. S4-6Response surface experimental results/WSDF-AB1.jpg]

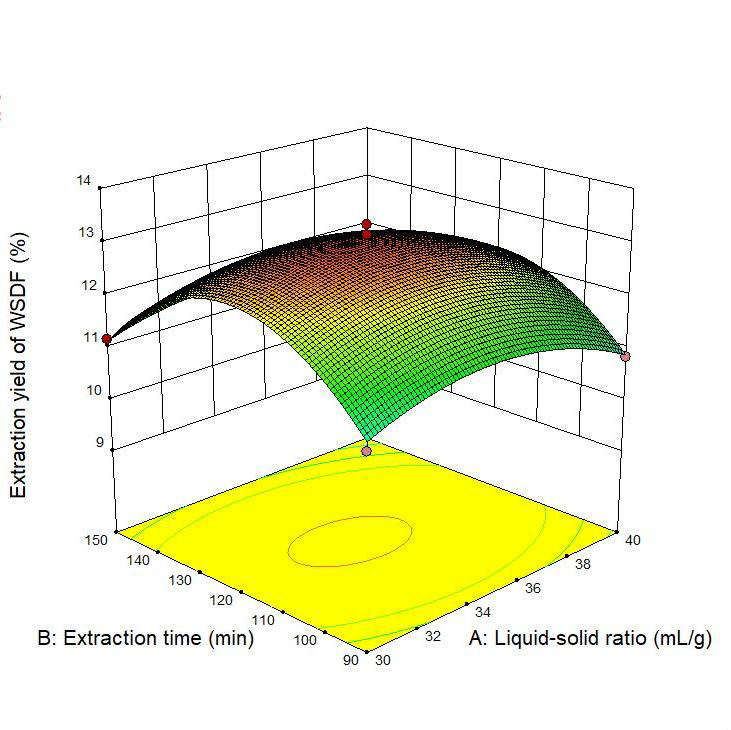

Supplement: Supplementary file 1 [file nutrients-16-03650-s001.zip › Fig. S4-6Response surface experimental results/WSDF-AB2.jpg]

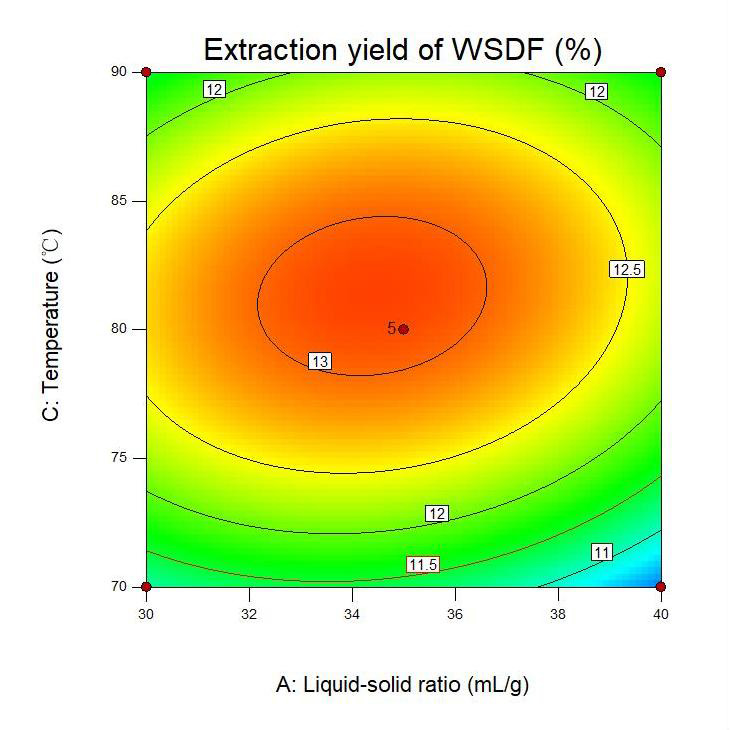

Supplement: Supplementary file 1 [file nutrients-16-03650-s001.zip › Fig. S4-6Response surface experimental results/WSDF-AC.jpg]

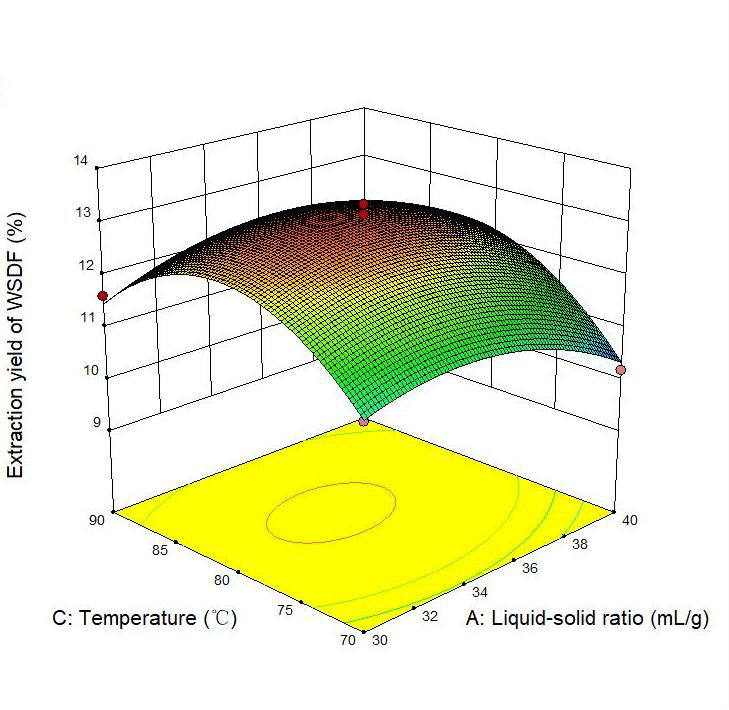

Supplement: Supplementary file 1 [file nutrients-16-03650-s001.zip › Fig. S4-6Response surface experimental results/WSDF-AC2.jpg]

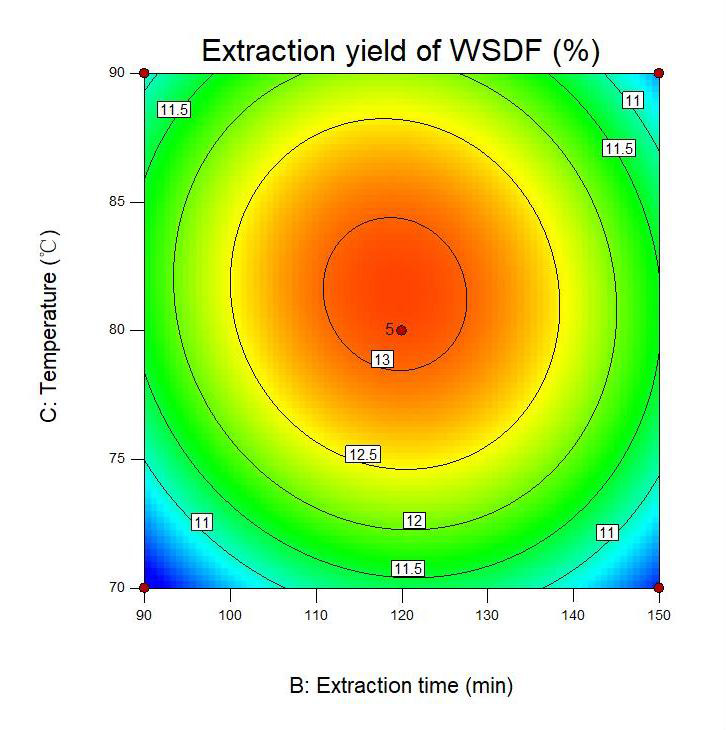

Supplement: Supplementary file 1 [file nutrients-16-03650-s001.zip › Fig. S4-6Response surface experimental results/WSDF-BC.jpg]

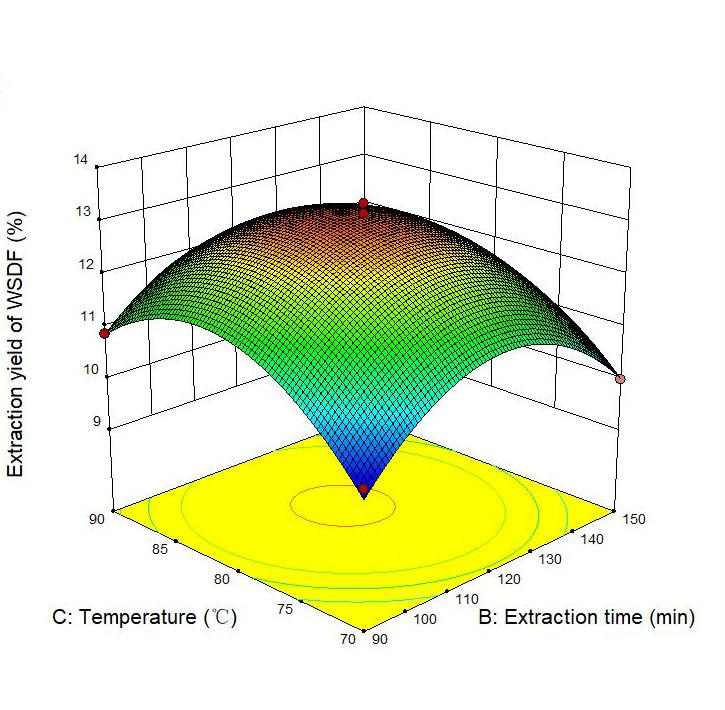

Supplement: Supplementary file 1 [file nutrients-16-03650-s001.zip › Fig. S4-6Response surface experimental results/WSDF-BC2.jpg]
